# Spotting disease disrupts the microbiome of infected purple sea urchins, *Strongylocentrotus purpuratus*

**DOI:** 10.1186/s12866-023-03161-9

**Published:** 2024-01-04

**Authors:** Chloe G. Shaw, Christina Pavloudi, Ryley S. Crow, Jimmy H. Saw, L. Courtney Smith

**Affiliations:** 1 Department of Biological Sciences, George Washington University, Washington, DC USA; 2 Present Address: European Marine Biological Resource Centre (EMBRC-ERIC), Paris, France

**Keywords:** Microbiome, Infection, Lesion, Pathogenic, 16S rRNA, Disease

## Abstract

**Background:**

Spotting disease infects a variety of sea urchin species across many different marine locations. The disease is characterized by discrete lesions on the body surface composed of discolored necrotic tissue that cause the loss of all surface appendages within the lesioned area. A similar, but separate disease of sea urchins called bald sea urchin disease (BSUD) has overlapping symptoms with spotting disease, resulting in confusions in distinguishing the two diseases. Previous studies have focus on identifying the underlying causative agent of spotting disease, which has resulted in the identification of a wide array of pathogenic bacteria that vary based on location and sea urchin species. Our aim was to investigate the spotting disease infection by characterizing the microbiomes of the animal surface and various tissues.

**Results:**

We collected samples of the global body surface, the lesion surface, lesioned and non-lesioned body wall, and coelomic fluid, in addition to samples from healthy sea urchins. 16S rRNA gene was amplified and sequenced from the genomic DNA. Results show that the lesions are composed mainly of Cyclobacteriaceae, Cryomorphaceae, and a few other taxa, and that the microbial composition of lesions is the same for all infected sea urchins. Spotting disease also alters the microbial composition of the non-lesioned body wall and coelomic fluid of infected sea urchins. In our closed aquarium systems, sea urchins contracted spotting disease and BSUD separately and therefore direct comparisons could be made between the microbiomes from diseased and healthy sea urchins.

**Conclusion:**

Results show that spotting disease and BSUD are separate diseases with distinct symptoms and distinct microbial compositions.

**Graphical abstract:**

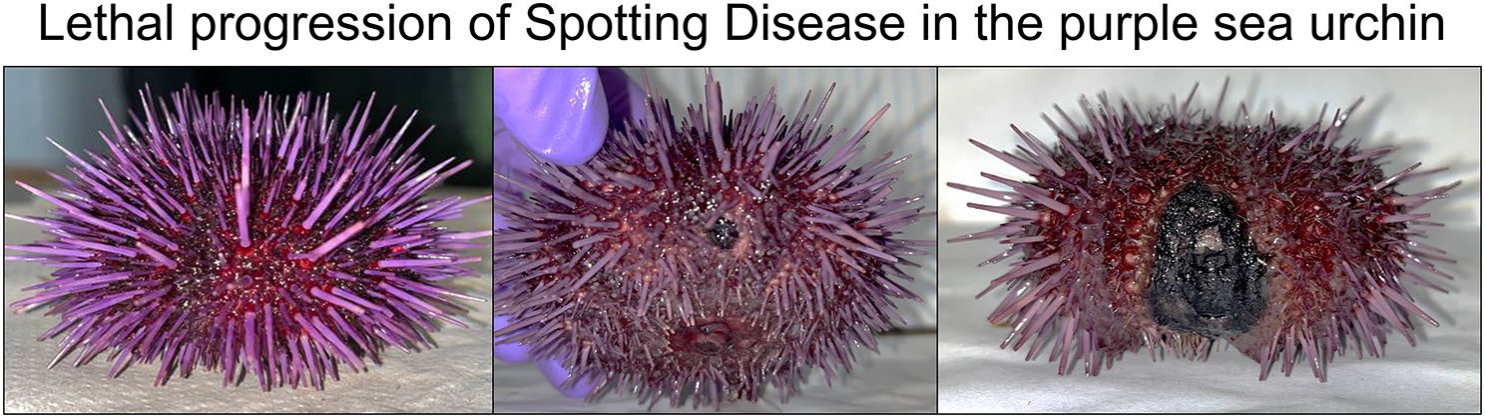

**Supplementary Information:**

The online version contains supplementary material available at 10.1186/s12866-023-03161-9.

## Background

Spotting disease, or red spotting disease, has been described for many sea urchin species and shows a range of symptoms based on both the species that are impacted and the severity of the disease [1–5]. Spotting disease is characterized by discrete viscous surface lesions composed of blackened necrotic tissue on the body wall that typically occur at the ‘equator’ of these spheroid animals and that result in a loss of all surface appendages within the lesion. Spotting disease requires injury to initiate the infection [6, 7], which can happen when the spines of neighboring sea urchins abrade or pierce the surface tissues. This is consistent with the location of lesions on the equator of the body. Lesions range in size from a few mm^2^ to more than a third of the body surface [8] and the infected tissue can be discolored, which includes green [8], blue-green/olive [9], dark red/purple [1], or black [2, 4, 5]. The lesions expand to infect more of the body surface and deepen to expose and subsequently degrade the test. Spotting disease lesions have been reproduced in a laboratory setting by mechanical abrasion, which has also been used to identify causative agents of spotting disease by infecting wounds with specific bacteria following the abrasion [2, 4, 6, 7]. Many bacterial species reproduce the discrete lesions and therefore there are multiple causative agents underlying spotting disease [2, 4, 5, 7]. Spotting disease is a major problem for sea urchins housed in aquaculture facilities because of the high density of the animals, as well as frequent handling and substandard culturing conditions that increase the frequency of disease [10], which results in significant economic loss [11]. Separation of sea urchins into individual spaces in aquaculture facilities significantly reduces the impact from diseases [12].

A separate disease of sea urchins, bald sea urchin disease (BSUD), also affects sea urchin species across many locations. BSUD has variable descriptions in the literature, but is generally characterized by a loss of appendages over most or all of the surface [13–15]. However, surface lesions may be present that degrade the epidermal tissue and can damage the muscle fibers at the base of the spines, in the tube feet and the associated ampullae [6, 8, 13, 14, 16–22]. Because BSUD is commonly described with necrotic lesions, which are characteristic of spotting disease, it is generally lethal because the lesions may perforate the test plate causing death [1, 8, 17, 18]. As a result, the descriptions of BSUD and spotting disease overlap, which results in significant confusion regarding the differences between these two diseases. Because we have reported changes in the microbiome on sea urchins infected with BSUD in our aquaria [15], and spotting disease occurred on animals in the same aquaria, our aim is to understand whether the microbiomes associated with these two diseases are different when the animals are housed under the same conditions.

Here, we describe the lesions and the microbiomes associated with spotting disease on purple sea urchins, *Strongylocentrotus purpuratus*, housed in closed, recirculating aquaria. Although previous studies have focused on identifying the causative agent underlying spotting disease, which suggests a wide array of pathogenic bacteria, few studies have reported an analysis of microbiomes of tissues from sea urchins infected with spotting disease [5]. Furthermore, none report the microbiome of tissues from sea urchins housed in a closed aquarium system, which have distinct properties and dynamics compared to open aquarium systems, leading to direct impacts on the host microbiome [23]. Because the microbiome on sea urchins shifts when transferred from the ocean to a closed aquarium [24], sampling sea urchins housed in the same aquarium system allows direct comparisons among the microbiome samples. We collected global surface samples, lesion surfaces, lesioned and non-lesioned body wall tissues, and coelomic fluid from diseased and healthy sea urchins, and the 16S rRNA gene was sequenced to characterize the microbiomes of each sample. Results indicate that the global surface microbiomes consisting of the microbes present on the external surface differ between diseased and healthy sea urchins, despite being housed in the same aquarium. Furthermore, the microbiomes differ between lesioned and non-lesioned body wall tissue sampled from diseased sea urchins, and between non-lesioned body wall tissue and coelomic fluid from diseased compared to healthy sea urchins. Results also show that the microbiomes associated with spotting disease are distinct from those associated with BSUD for sea urchins housed in the same aquarium. This suggests that spotting disease and BSUD are different diseases and that this difference correlates with discrete lesions that are characteristic of spotting disease but not BSUD.

## Materials and methods

### Sea urchin husbandry

Purple sea urchins, *Strongylocentrotus purpuratus*, were purchased from the Southern California Sea Urchin Company (Corona del Mar, CA) and shipped to George Washington University in Washington DC. Sea urchins were housed in a 125 gallon aquarium (aquarium B) with recirculating artificial seawater (Premium Marine Salt, OmegaSea), salinity of 32–35 ppt, 13–14℃, and outfitted with both physical and bio-filters, a UV light housing, and a protein skimmer. The central aquarium pump (Pond-Mag 9.5, Pondmaster) that was positioned in the aquarium sump, circulated 950 gallons/hour through the system. The water quality was maintained with weekly seawater changes of 5 gallons that also served in solid waste removal. All animals were fed weekly with rehydrated brown seaweed, *Saccharina angustata* (Kjellman) (WEL-PAC). Diseased sea urchins were maintained in individual plastic floating holding boxes to prevent interaction with healthy sea urchins and to monitor disease progression.

### Treatment of sea urchins with penicillin and streptomycin

Based on our standard protocol, all shipments of sea urchins were treated upon arrival by immersion for 1–2 h at 14℃ in a tray (8 L) of freshly prepared artificial seawater with 12 mg/L penicillin and 50 mg/L streptomycin sulfate (pen/strep). After treatment, sea urchins were returned to their aquarium.

### Sample collection

#### Surface microbiome

To collect samples from the global surface microbiomes, diseased sea urchins (n = 4; D1-D4, Table 1) and healthy sea urchins (n = 4; H1-4) from aquarium B were placed in a funnel connected to a microbial collection system as described previously [15]. Briefly, seawater (500 ml) from aquarium B was poured onto the ventral side of the sea urchins, so that the seawater washed over the entire body and the cellular material was collected on nylon filters (0.22 𝜇m, 47 mm diameter; GVS Filter Technology) held on a filter-holder assembly. Seawater samples (500 ml, n = 2) (fSW) were filtered in the same manner and served as controls. A sample of 500 ml of freshly mixed OmegaSea seawater (foSW) was also filtered to serve as the negative control. The nylon filters were stored in 50 ml falcon tubes at -80℃ for later use.

**Table 1:** Definitions of sample abbreviations

Group	Sample abbreviation	Definition of abbreviations	Sample type
LS	D1LS	Diseased sea urchin 1 lesion surface	Swab
	D2aLS	Diseased sea urchin 2 lesion a - lesion surface	Swab
	D2bLS	Diseased sea urchin 2 lesion b - lesion surface	Swab
	D3LS	Diseased sea urchin 3 lesion surface	Swab
	D4LS	Diseased sea urchin 4 lesion surface	Swab
LBW	D1LBW1	Diseased sea urchin 1 lesioned body wall	Dissected tissue
	D1LBW2	Diseased sea urchin 1 lesioned body wall (replicate)	Dissected tissue
	D2aLBW1	Diseased sea urchin 2 lesion a - lesioned body wall	Dissected tissue
	D2aLBW2	Diseased sea urchin 2 lesion a - lesioned body wall (replicate)	Dissected tissue
	D2bLBW1	Diseased sea urchin 2 lesion b - lesioned body wall	Dissected tissue
	D2bLBW2	Diseased sea urchin 2 lesion b - lesioned body wall (replicate)	Dissected tissue
	D3LBW	Diseased sea urchin 3 lesioned body wall	Dissected tissue
DBW	D1BW1	Diseased sea urchin 1 non-lesioned body wall	Dissected tissue
	D1BW2	Diseased sea urchin 1 non-lesioned body wall (replicate)	Dissected tissue
	D2BW1	Diseased sea urchin 2 non-lesioned body wall	Dissected tissue
	D2BW2	Diseased sea urchin 2 non-lesioned body wall (replicate)	Dissected tissue
	D3BW1	Diseased sea urchin 3 non-lesioned body wall	Dissected tissue
	D3BW2	Diseased sea urchin 3 non-lesioned body wall (replicate)	Dissected tissue
DCF	D1CF	Diseased sea urchin 1 coelomic fluid	Coelomic fluid
	D2CF	Diseased sea urchin 2 coelomic fluid	Coelomic fluid
	D3CF	Diseased sea urchin 3 coelomic fluid	Coelomic fluid
HBW	H1BW1	Healthy sea urchin 1 non-lesioned body wall	Dissected tissue
	H1BW2	Healthy sea urchin 1 non-lesioned body wall (replicate)	Dissected tissue
	H2BW1	Healthy sea urchin 2 non-lesioned body wall	Dissected tissue
	H2BW2	Healthy sea urchin 2 non-lesioned body wall (replicate)	Dissected tissue
	H3BW1	Healthy sea urchin 3 non-lesioned body wall	Dissected tissue
	H3BW2	Healthy sea urchin 3 non-lesioned body wall (replicate)	Dissected tissue
HCF	H1CF	Healthy sea urchin 1 coelomic fluid	Coelomic fluid
	H2CF	Healthy sea urchin 2 coelomic fluid	Coelomic fluid
	H3CF	Healthy sea urchin 3 coelomic fluid	Coelomic fluid
Controls	fSW1	500 ml filtered seawater from aquarium B	Aquarium seawater
	fSW2	500 ml filtered seawater from aquarium B	Aquarium seawater
	foSW	500 ml filtered Omega seawater	Seawater
	sSW	Swab of seawater from aquarium B	Swab

#### Swab collections

Diseased sea urchins (n = 4) were rinsed with 500 ml 0.22 𝜇m filtered artificial seawater (faSW) that was poured directly onto the ventral side of the animals such that the seawater washed over the body surface and removed microbes that were not tightly associated with the lesions. Sterile swabs were used to collect cells from the lesion surface (LS) with gentle rotation and twisting until the swab tip was coated with material. The coated swab was inserted into a 50 ml falcon tube filled with 35 ml faSW, and the swab stick end was removed using sterile scissors. A sterile swab was dipped into the seawater in aquarium B and inserted into a 50 ml falcon tube to collect sample microbes in the aquarium seawater (sSW). The falcon tubes containing swabs were vortexed vigorously to release the microbes, and the contents were poured into the vacuum filtration system to transfer the cells to the nylon 0.22 𝜇m filters, as described above. An additional 20 ml of faSW was added to the falcon tube with the swabs, vortexed vigorously, and poured into the vacuum filtration system. Filters were stored in 50 ml falcon tubes at -80℃.

#### Sea urchin sacrifice for tissue collection

Surviving diseased sea urchins (n = 3) and healthy sea urchins (n = 3) were rinsed with 500 ml faSW, as described above. Sea urchins were sacrificed by cutting the peristomial membrane around Aristotle’s lantern with sterile scissors and removing the lantern. The whole coelomic fluid (CF, cells and fluid) from each animal was poured into a 50 ml falcon tube (diseased CF, DCF; healthy CF, HCF). For diseased animals, tissue samples of lesioned body wall (LBW, n = 7) and non-lesioned body wall (DBW, n = 6) were collected by cutting through the body wall with sterile scissors and sterile forceps. Non-lesioned areas of the body wall (HBW, n = 6) were also collected from healthy animals (H1-H3) by the same method. Replicate samples were collected for each tissue except for D3LBW (the entire lesion was collected in the first sample), and the CF samples (all of the coelomic fluid was collected for each sea urchin). Tissue samples were placed in 50 ml falcon tubes filled with 35 ml of ice chilled faSW and ground with glass rods and vortexed vigorously, whereas the coelomic fluid was allowed to clot on ice. Tissue samples were centrifuged at 4300 x *g* for 5 min at 4℃, and the supernatant was poured into the vacuum system to collect the microbes on 0.22 𝜇m filters, as described above. Additional faSW (40 ml) was added to each pelleted tissue sample, vortexed vigorously, centrifuged, and the supernatant collected on filters. This process was repeated once. Filters were stored in 50 ml falcon tubes at -80℃.

### Genomic DNA isolation from samples

The genomic (g)DNA isolation from nylon filters was carried out according to Turner et al. [25] with modifications described by Shaw et al. [15]. Briefly, filters were incubated with 1 ml cetyltrimethylammonium bromide (CTAB; 2% CTAB, 100 mM Tris pH 7.4, 4 M NaCl, 1% polyvinylpyrrolidone, 20 mM EDTA), to which was added 1 ml of chloroform:isoamyl alcohol (24:1), precipitated with 2.5 M NaCl and 50% isopropanol, and resuspended in Tris-EDTA buffer (TE; 10 mM Tris base pH 7.4, 1 mM EDTA). The gDNA concentration was evaluated on a spectrophotometer (NanoDrop 2000c, ThermoFisher). The gDNA size and level of degradation was evaluated with a 0.75% agarose gel with Tris-acetate-EDTA buffer (TAE; 40 mM Tris; 20 mM acetic acid, 1 mM EDTA) plus ethidium bromide and imaged with a UV imaging system (Kodak Molecular Imaging, Kodak Gel Logic 1500 Imaging System).

### Polymerase chain reactions

To verify that bacterial gDNA was present in the samples prior to sequencing, the 16S rRNA gene was amplified with one of two sets of test primers; either 27 F (AGA GTT TGA TCC TGG CTC AG) and 1492R (ACG GTT ACC TTG TTA CGA CTT) [26], or 331 F (TCC TAC GGG AGG CAG CAG T) and 797R (GGA CTA CCA GGG TAT CTA ATC CTG TT) [27]. The PCR reaction of 20 μl contained 1X ExTaq Buffer, 200 μM dNTPs, 0.5 or 1 μM each primer, and 0.5 units Ex Taq DNA polymerase (Takara). The two sets of primers were used in different PCR programs. The step-down program for the 27 F/1492R primers was 94℃ for 30 s, followed by 4 cycles of 98℃ for 10 s, 57.4℃ for 30 s and 72℃ for 90 s, then 4 cycles of 98℃ for 10 s, 55.5℃ for 30 s and 72℃ for 90 s, followed by 17 cycles of 98℃ for 10 s, 53.7℃ for 30 s and 72℃ for 90 s, with a final extension of 72℃ for 2 min and a 4℃ hold. The step-down program for the 313 F/797R primers was 94℃ for 30 s followed by 4 cycles of 98℃ for 10 s, 58℃ for 30 s, 72℃ for 30 s, with a -1℃ change in annealing temperature per cycle to 55^o^C, then 21 cycles of 98℃ for 30 s, 54℃ for 30 s and 72℃ for 30 s, finally 72℃ for 2 min, and a 4℃ hold. The amplicons were analyzed on a 0.8% or 1% agarose gel with TAE buffer plus ethidium bromide and imaged on an UV system as described above.

### 16S rRNA amplicon sequencing

The gDNA samples were processed and sequenced using the ZymoBIOMICS targeted sequencing service at ZymoResearch (Irvine CA). The targeted sequencing of the bacterial 16S rRNA gene was carried out as described [15], using the *Quick*-16S NGS Library Prep Kit (ZymoResearch) with custom-designed primers to amplify the V3-V4 region of the 16S rRNA gene, real-time PCR and qPCR fluorescence readings, the Select-a-Size DNA Clean & Concentrator (ZymoResearch), Tapestation (Agilent Technologies) and Qubit (ThermoFisher Scientific). The positive control sample used for library preparation was the ZymoBIOMICS Microbial Community DNA Standard (ZymoResearch). The negative controls included the foSW sample as a control for the DNA extraction method, and a blank for library preparation that was provided by ZymoResearch. The completed library was sequenced using a V3 reagent kit (600 cycles) on Illumina MiSeq, which was calibrated by a 10% spike-in of PhiX DNA. The raw sequence reads were uploaded to the Sequence Read Archive database at NCBI under the BioProject ID PRJNA937707.

### Amplicon sequence analysis

Amplicon sequence analysis was carried out as reported [15] using the DADA2 pipeline [28], SILVA release 138.1 for taxonomy [29] and the Phyloseq package (ver 1.42.0) [30]. Beta diversity was analyzed using weighted unique fraction (UniFrac) and visualized using non-metric multidimensional scaling (NMDS). Statistical analysis and Linear Discriminant Analysis Effect Size (LEfSe) were also carried out as previously reported, by linear discriminant analysis (LDA) cutoff set to 2, and all analyses were performed using R version 4.1.1 [31]. The R code with the complete pipeline can be found in the GitHub repository Spotting_disease_S_purpuratus.

## Results

### Spotting disease progression is similar for all infected sea urchins

**Fig. 1:**
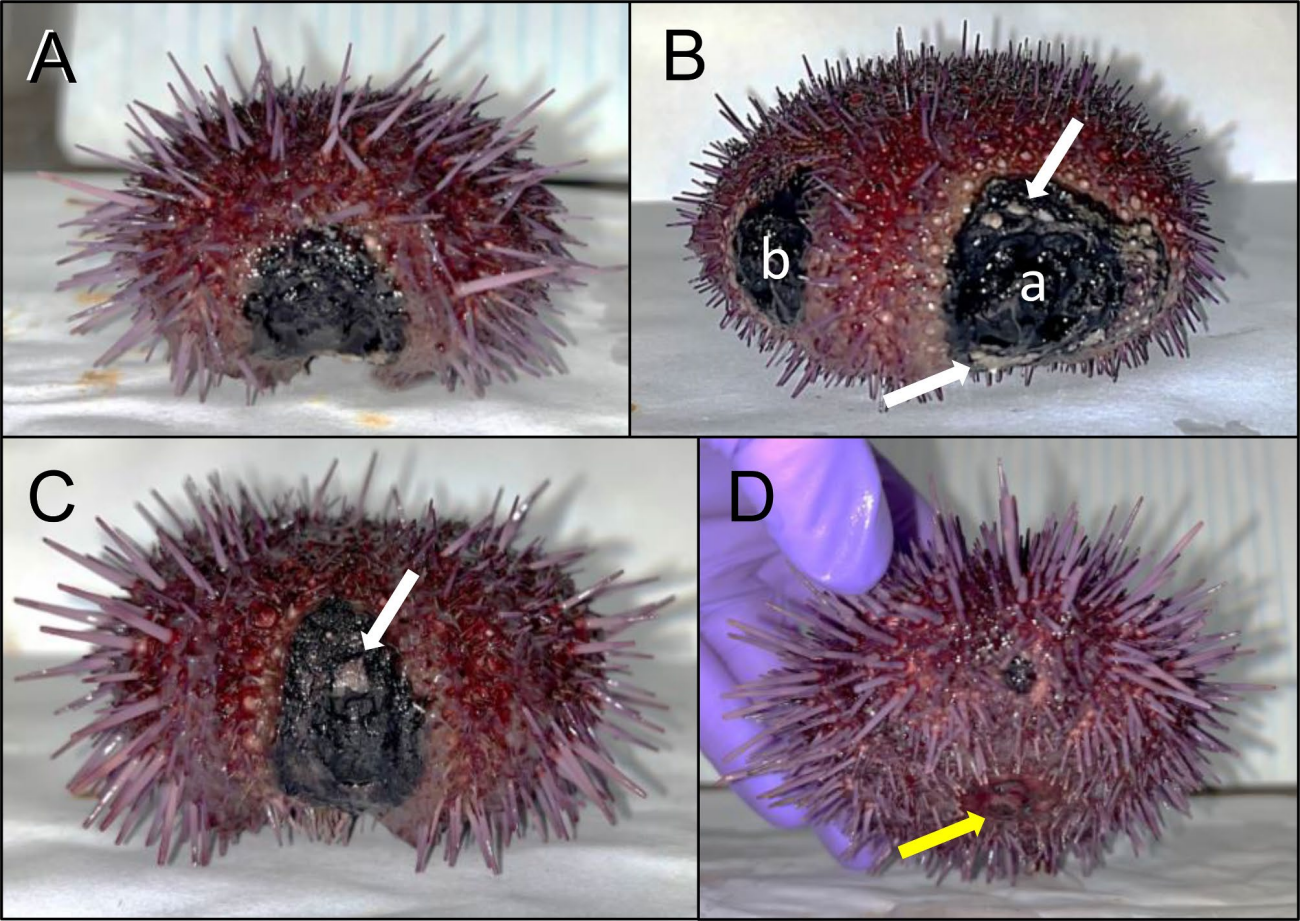
Sea urchins with spotting disease show discrete lesions at the equatorial to ventral body regions. All sea urchins are positioned with the oral surface facing down. **A** Diseased sea urchin (D1) has a single large black necrotic lesion. This sea urchin displays unusual orientation of its spines, which point in various directions rather than uniformly perpendicular to the body surface. This is a behavioral indication of disease that has been noted previously [15]. **B** Diseased sea urchin (D2) has two large black necrotic lesions, labelled “a” and “b”. Parts of the test (white) are exposed around the outer region of lesion a (arrows). This sea urchin has lost all primary spines, including non-lesioned areas of the body surface, which indicates its moribund condition. **C** Diseased sea urchin (D3) has one large black necrotic lesion. This sea urchin shows typical spine orientation of perpendicular to the body surface, which indicates better health despite the lesion. Exposed test is also evident within the lesion (arrow). **D** Diseased sea urchin (D4) has a small black necrotic lesion of approximately 1 cm in diameter. The mouth is located on the ventral side (yellow arrow). This sea urchin also shows indications of better health, including primary spines generally pointing perpendicular to the body surface

While being housed in our aquaria, a small number of sea urchins contracted spotting disease, which allowed us to observe disease progression in a controlled environment. Diseased sea urchins showed one or two discrete regions of blackened necrotic tissue of varying sizes (Fig. 1). For all infected sea urchins, progression of the infection was similar. Initially the lesions were small and nearly undetectable; however, as the infection continued, the epidermal tissue was degraded, resulting in the loss of all appendages within the lesioned area (Fig. 1). When the lesions expanded and deepened, the underlying test was exposed, and sea urchins lost the primary spines in areas surrounding the lesion. Eventually, the test disintegrated, which signaled that a sea urchin would succumb soon afterwards, indicating that spotting disease was fatal. The duration of infection typically lasted months to years, however, the final phase of infection after the test was degraded tended to develop rather quickly as a sea urchin became moribund. In this late stage of the disease, sea urchins demonstrated altered behaviors, which, in addition to primary spine loss in non-lesioned regions, included cessation of eating and failing to hold on to anything with their tube feet (i.e., aquarium wall, kelp, holding box). In all cases, infected sea urchins never showed signs of recovery from spotting disease. Spotting disease was not communicable, as illustrated when sea urchins with lesions were housed in the same aquarium with healthy sea urchins, and the disease never appeared on the healthy animals.

The four infected sea urchins were sampled at different stages of spotting disease as inferred from the different sizes and numbers of lesions (Fig. 1). Diseased animal 1 (D1) had one large lesion and at the time of sacrifice, it was not eating consistently, which was an indication of impending mortality (Fig. 1A). Diseased animal 2 (D2) had two lesions of different sizes that were referred to as ‘a’ and ‘b’ for the larger and smaller lesions, respectively (Fig. 1B). At the time of sacrifice, D2 had lost all primary spines, was no longer feeding, and did not display tube foot function to attach to the box in which it was housed, all of which were indicators of its moribund state. Diseased animal 3 (D3) had one large lesion but exhibited normal tube foot function, all spines were present except for those surrounding the lesion, and it was eating normally (Fig. 1C). Sea urchins D1, D2, and D3 were all treated twice with pen/strep; once at half concentration, and a second time at the standard concentration, one week apart. The pen/strep treatments had no effect on resolving the discrete lesions. Diseased animal 4 (D4) had one very small lesion that was first noticed once it was approximately 1 cm in diameter (Fig. 1D). D4 did not show any other signs of altered behaviors associated with spotting disease. Unfortunately, this animal died due to unrelated factors and therefore was not used in the analysis of dissected tissues that were collected from the other sea urchins upon sacrifice. However, D4 samples were collected for the analysis of the global surface microbiome and LS.

### Sequence data identifies ASVs in all samples

Preliminary analyses by PCR of the microbial community gDNA isolated from the samples of both diseased and healthy sea urchins confirmed that bacterial gDNA was present in in all samples. 16S rRNA gene sequencing resulted in a total of 81,536 non-chimeric reads (Table 2).

**Table 2:** Read processing summary

Sample Name*	Initial reads	Filtered reads	Forward denoised reads	Reverse denoised reads	Merged reads	Non-chimeric reads
D1 surface	33,726	14,952	14,752	14,917	931	797
D2 surface	17,595	16,723	16,526	16,551	799	723
D3 surface	33,384	32,146	31,755	31,637	1817	1271
D4 surface	48,328	45,818	45,501	45,593	1821	1629
H1 surface	35,032	33,574	33,273	33,170	2402	1809
H2 surface	46,959	24,152	23,455	23,929	852	675
H3 surface	35,605	33,855	33,658	33,530	4404	2571
H4 surface	47,456	45,630	45,424	45,132	7623	3265
fSW1	31,305	12,132	11,862	12,077	901	756
fSW2	37,718	16,042	15,462	15,957	789	689
D1LS	35,469	33,859	33,731	33,722	2345	2092
D2aLS	42,909	41,188	40,991	40,858	2218	1929
D2bLS	45,379	43,625	43,536	43,418	2208	1935
D3LS	49,980	21,694	21,395	21,632	1070	896
D4LS	37,435	35,707	35,542	35,585	432	376
sSW	48,680	20,150	19,895	19,810	727	473
D1LBW1	1184	1128	1010	756	2	2
D1LBW2	45,357	44,356	44,215	44,152	5074	3846
D1BW1	36,896	35,314	35,256	35,155	2668	2095
D1BW2	40,374	38,793	38,738	38,593	4921	3011
D1CF	2251	850	838	846	28	26
D2aLBW1	41,654	20,656	20,556	20,426	300	268
D2aLBW2	41,248	39,378	39,335	39,142	1272	975
D2bLBW1	56,938	28,786	28,624	28,455	1678	1412
D2bLBW2	44,766	20,958	20,746	20,801	1358	1115
D2BW1	44,633	42,758	42,713	42,560	4011	1995
D2BW1	47,160	45,031	44,962	44,843	3203	1810
D2CF	45,370	42,968	42,906	42,772	1569	1122
D3LBW1	56,282	54,004	53,949	53,760	978	843
D3BW1	39,944	18,742	18,226	18,700	2827	1299
D3BW2	2704	2630	2604	2585	4	2
D3CF	58,380	56,053	55,982	55,422	20,583	2755
H1BW1	48,161	17,693	17,023	17,685	1803	1773
H1BW2	62,699	60,458	60,385	59,924	26,964	13,253
H1CF	63,084	60,727	60,492	60,422	9486	8717
H2BW1	53,428	22,004	21,742	21,963	1565	1253
H2BW2	48,533	22,693	22,316	22,630	1448	1108
H2CF	40,126	17,722	17,439	17,582	373	279
H3BW1	54,753	52,648	52,511	52,475	5782	4393
H3BW2	75,964	37,381	36,890	37,242	3972	3140
foSW	50,319	14,087	14,038	14,084	445	258
Total	1,810,880	1,347,029	1,337,510	1,337,723	137,291	81,536

*See Table 1 for sample name definitions

### The global surface microbiomes of diseased sea urchins show taxonomic differences compared to the global surface microbiomes of healthy sea urchins

**Fig. 2:**
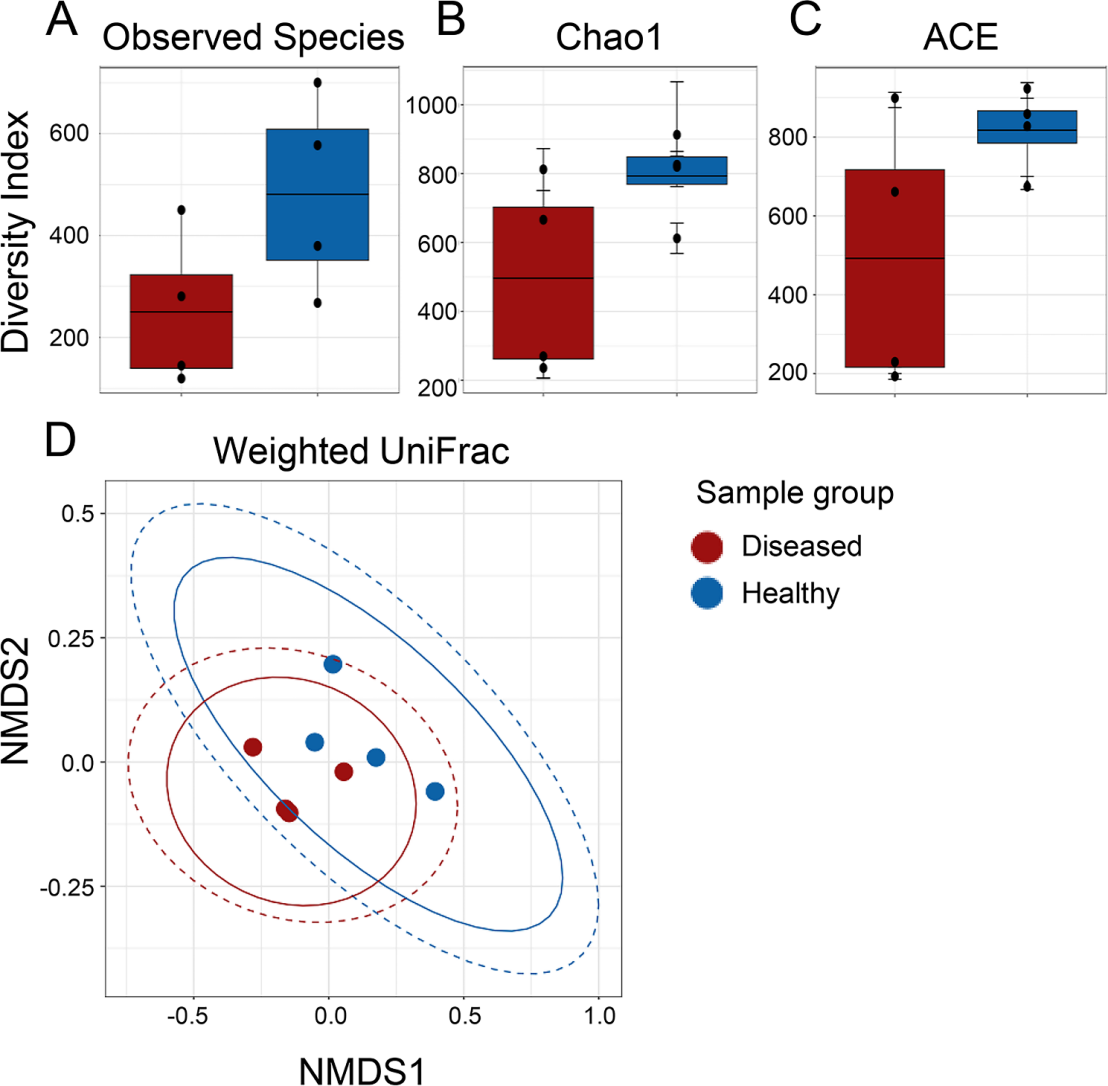
The global surface microbiome diversity is not different between diseased and healthy sea urchins. Alpha diversity is analyzed by **A** Observed Species, **B** Chao1, and **C** ACE. The box plots show the mean and quartile values for each group, which are not significantly different (ANOVA, *p* > 0.05). **D** Beta diversity is analyzed at the level of ASV sequences using weighted UniFrac and visualized with NMDS. Ellipses around sample groups show 95% confidence intervals assuming a multivariate t-distribution (solid line) or a multivariate normal distribution (dashed line). The beta diversity of the groups is not significantly different (PERMANOVA, *p* > 0.05), indicating that microbial composition is similar for the global surface microbiomes on diseased and healthy sea urchins housed in the same aquarium

The global surface microbiomes on echinoids are poorly studied. However, an initial report shows that they are altered when variegated sea urchins, *Lytechinus variegatus*, are transferred from open water to a closed system [24]. The surface microbiomes also shift as sea urchins recover from BSUD, and it can differ on sea urchins housed in different aquaria [15]. Because there were healthy sea urchins in the same aquarium with spotting disease, and the disease was not communicable, we investigated the global surface microbiome to identify differences between diseased and healthy sea urchins. The alpha diversity of the global surface microbiomes on diseased and healthy sea urchins were analyzed using observed species, Chao1 [32], and ACE [33] (Fig. 2A-C). Results showed that there were no significant differences in alpha diversity between the global surface microbiomes on the diseased compared to the healthy sea urchins (ANOVA, *p* > 0.05), although the diseased group microbiome had decreased alpha diversity compared to the healthy group for all metrics. Beta diversity was analyzed by weighted UniFrac that measures microbiome composition, which showed overlap of the samples from diseased and healthy sea urchins (PERMANOVA, *p* > 0.05; Fig. 2D), indicating that the microbiome composition was not significantly different between the two groups.

**Fig. 3:**
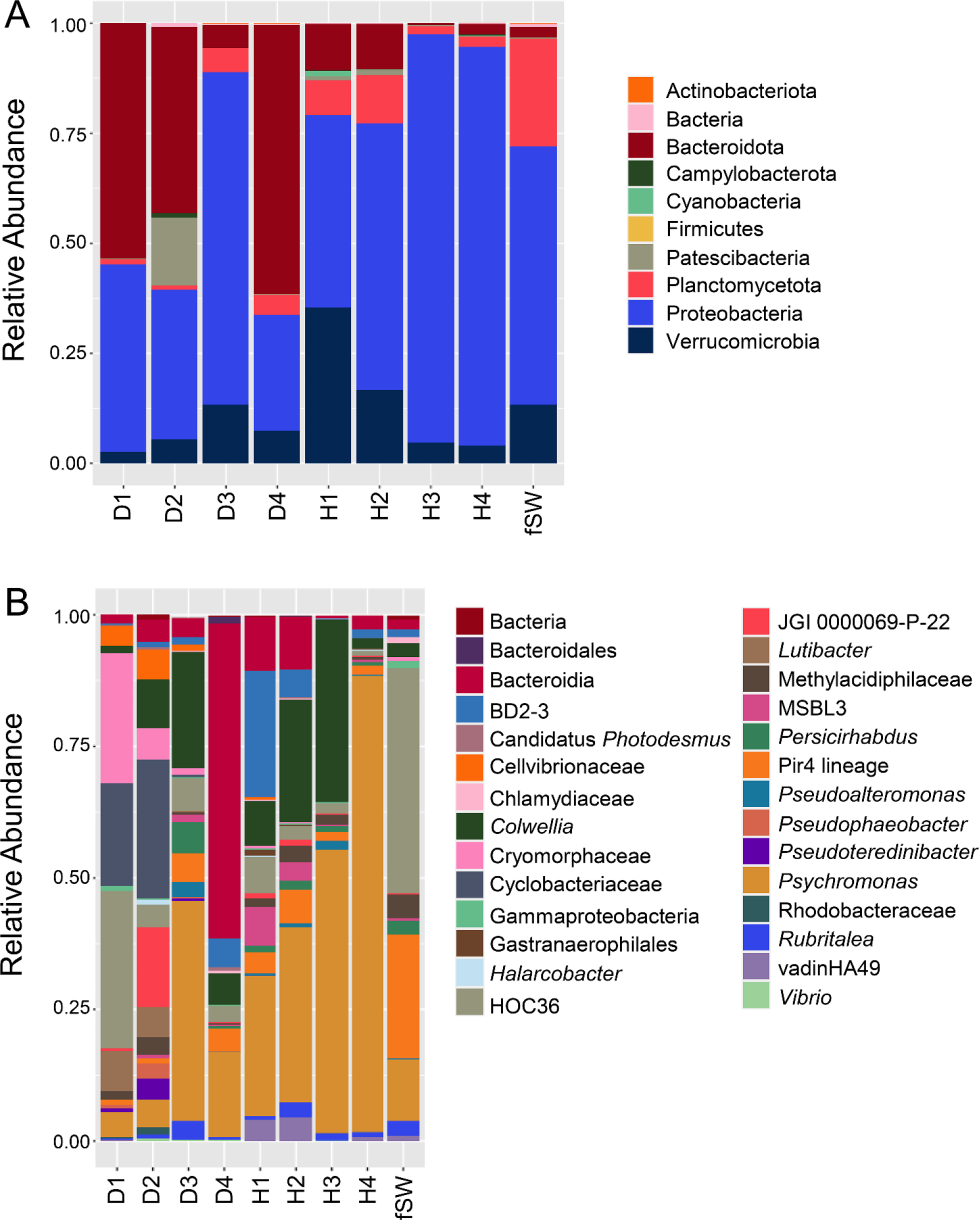
Global surface microbiomes of diseased vs. healthy sea urchins show taxonomic differences. **A** All identified phyla for the sea urchin surface samples and the fSW sample (Additional File, Table S1) illustrate the relative abundance of each taxon in each sample. **B** Genera with an average relative abundance of > 0.1% across all samples (Additional File, Table S2) are illustrated by the relative abundance per sample. fSW is the average of fSW1 and fSW2. Taxa in **A** and **B** that could not be assigned at the level of phylum or genus are listed as the most specific known taxonomic level. BD2-3 is in the order Victivallales, the Pir4 lineage is in the family Pirellulaceae, vadinHA49 is in the phylum Planctomycetota, JGI-0000069-P22 is in the class Gracilibacteria, MSBL3 is in the family Kiritimatiellaceae, and HOC36 is in the class Gammaproteobacteria. ASV sequences that could not be assigned to a phylum are grouped as Bacteria. Sample name abbreviations are defined in Table 1

**Fig. 4:**
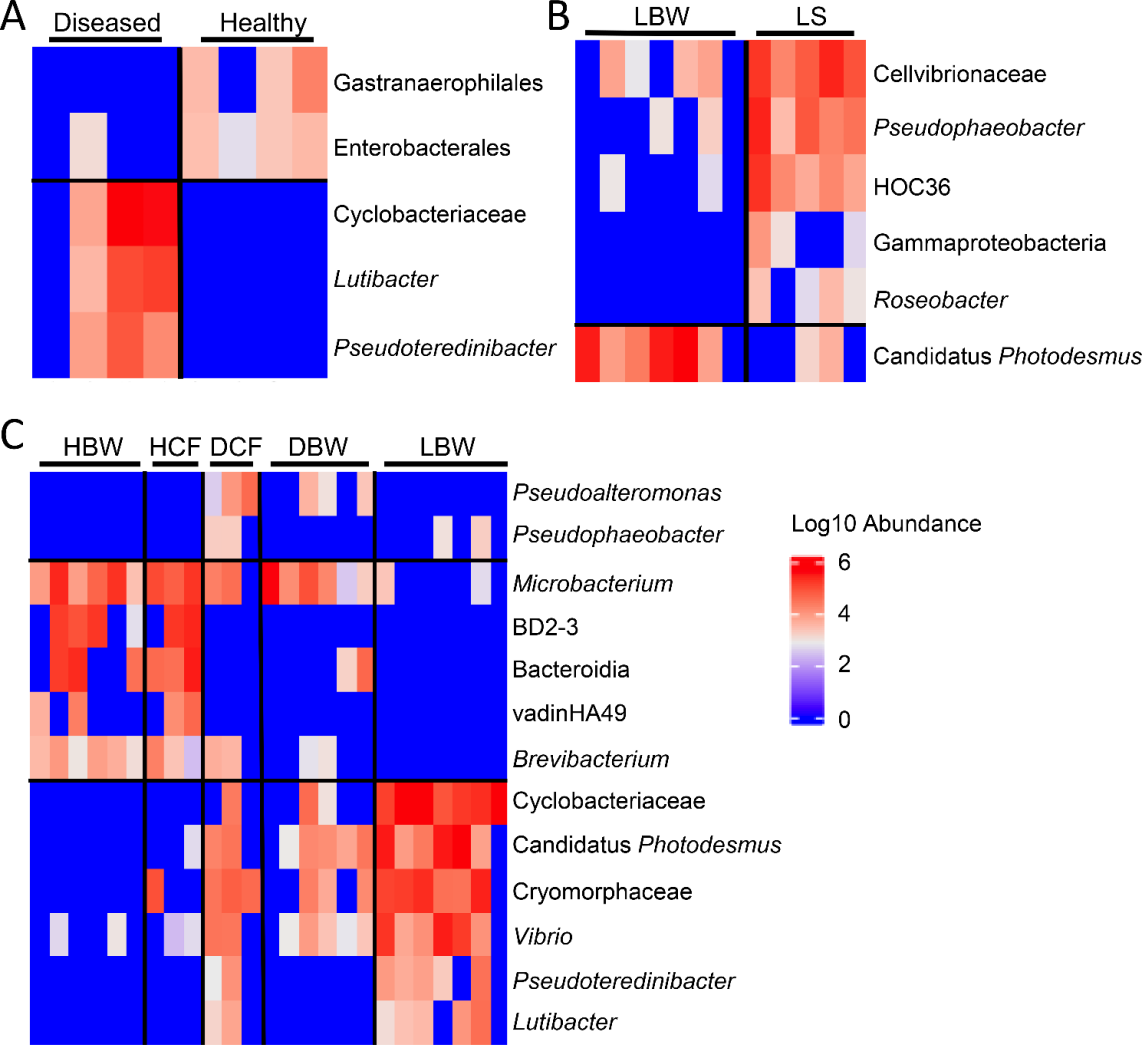
Many taxa are differentially abundant in the microbiome samples. A heatmap shows all taxa identified by LEfSe that are significantly differentially abundant (*p* < 0.05) and have an LDA score of > 2 as their abundance per group for **A** the global surface microbiome samples, **B** the lesioned body wall (LBW) and lesion surface (LS) microbiome samples, and **C** the tissue microbiome samples (Additional File, Tables S3, S6, S9). Sample name abbreviations are defined in Table 1

The composition of bacterial taxa collected from the two groups of sea urchins were compared to identify differences among the global surface microbiomes, in addition to comparisons with the two samples of filtered aquarium seawater (fSW). The most abundant taxa were selected (Additional File; Tables S1, S2) to identify the similarities and differences. Although beta diversity showed no differences among the samples (Fig. 2D), the phyla present in the global surface microbiomes were different between the diseased and healthy sea urchins (Fig. 3). Samples were generally dominated by Proteobacteria, and three of the diseased surface microbiome samples had an elevated abundance of the phylum Bacteroidota compared to the healthy surface microbiome samples (Fig. 3A). There were also major differences in the genera identified in the global surface microbiomes among the groups (Fig. 3B). The microbial composition of the diseased surface microbiome samples differed from the healthy surface microbiome samples, and there were also differences among the samples within the diseased group. The samples collected from D1 and D2 had many taxa in common with elevated abundances of a genus in the Cryomorphaceae family, a genus in the Cyclobacteriaceae family, *Lutibacter*, and a genus in the Cellvibionaceae family. This was not evident in the global surface samples from D3 and D4 nor from the corresponding samples from the healthy sea urchins. The D3 and D4 surface samples were more similar to the healthy surface samples, and were mainly composed of *Psychromonas*, *Colwellia*, and a family in the Bacteroidia class. Samples from the fSW were distinct from all samples collected from the sea urchins. It is notable that sea urchins D3 and D4, with less extensive lesions, had global surface microbiomes that were more similar to those on the healthy sea urchins, whereas D1 and D2, with more extensive lesions, had microbiomes that were very different from the healthy sea urchins. LEfSe analysis showed that Cyclobateriaceae, *Lutibacter* and *Pseudoteredinibacter* were significantly differentially abundant in the diseased global surface microbiome samples, and that Gastranaerophilales and Enterobacterales were significantly differentially abundant in the corresponding samples from the healthy sea urchins (Fig. 4A; Additional File, Table S3). These results showed that the microbial composition of the global surface microbiome differed in samples from sea urchins that were in different stages of spotting disease progression. Results also showed that taxonomic differences were evident for the genera identified in the global surface microbiomes of the diseased compared to the healthy groups of the sea urchins that were housed in the same aquarium, and that they were also different from the microbes in the fSW samples. These results indicated that spotting disease could be characterized based on the differences in the global surface microbiomes of diseased compared to healthy sea urchins irrespective of the influences of the bacterial composition of the seawater in the aquarium and did not require direct sampling of the lesions.

### The lesions have a unique microbial composition

**Fig. 5:**
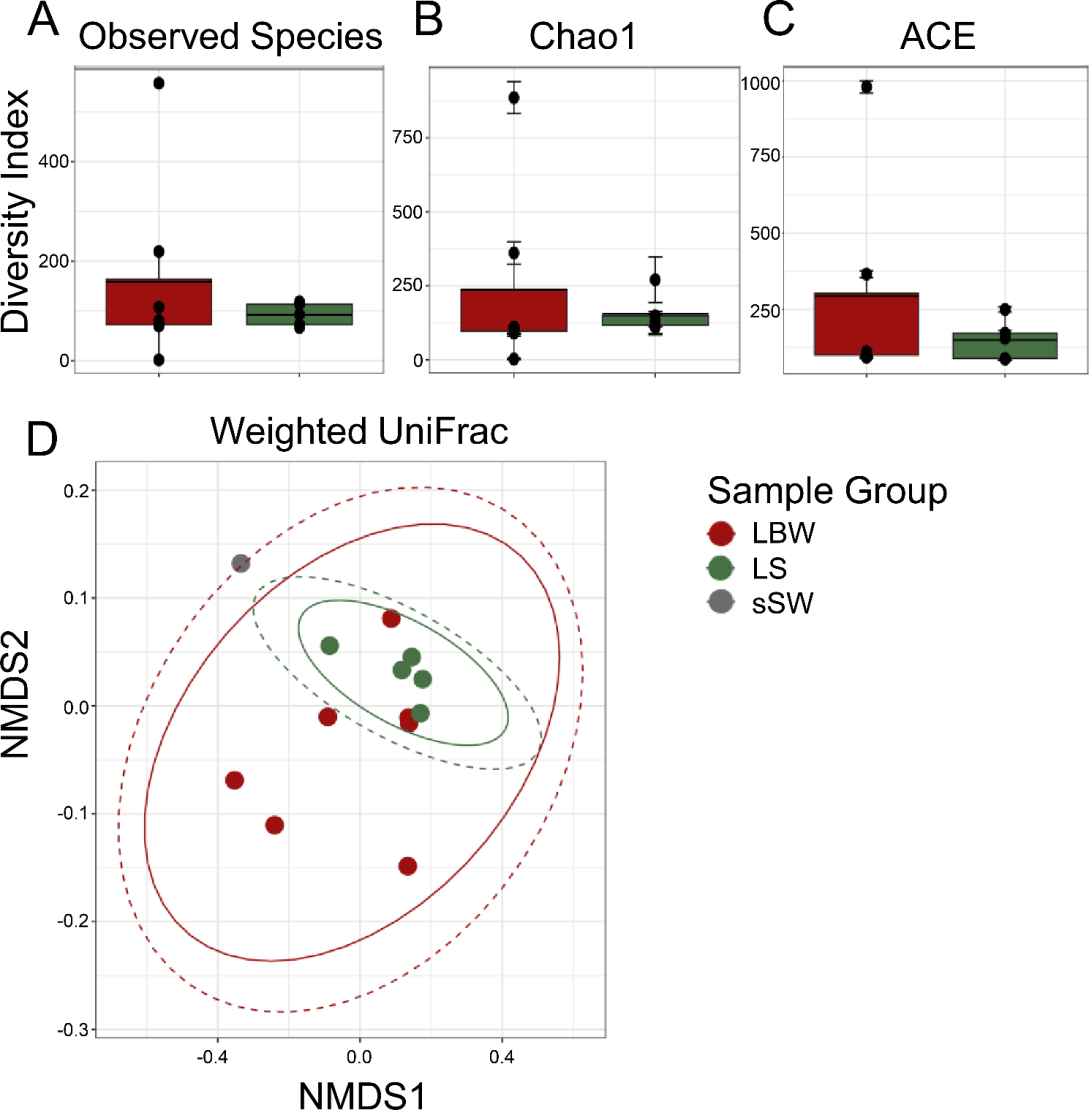
The microbiome compositions are similar between the lesion surface and the lesioned body wall. Alpha diversity of the microbiomes from the LS and LBW sample groups are evaluated by **A** Observed Species, **B** Chao1, and **C** ACE. The box plots show the mean and quartile values for each group, which are not different (ANOVA, *p* > 0.05). **D** Beta diversity is evaluated at the ASV level using weighted UniFrac and visualized with NMDS. Ellipses around sample groups show 95% confidence intervals assuming a multivariate t-distribution (solid line) and a multivariate normal distribution (dashed line). The microbiomes of the LS compared to the LBW sample groups are not significantly different (PERMANOVA, *p* > 0.05). The sSW sample is shown for comparison. Sample name abbreviations are defined in Table 1

One of the reported causative agents of spotting disease, *Vibrio coralliilyticus*, forms biofilms on the surface of tissues [4]. During the formation of a biofilm, bacteria aggregate, function as a single unit, and produce an extracellular matrix that protects the bacterial community from external factors such as antimicrobial agents [34]. Based on the appearance of the lesions on infected sea urchins and based on the lack of response to the pen/strep treatment by the diseased sea urchins, microbes associated with lesions may have formed a biofilm. To investigate this possibility, the LS was collected by the swab method to determine whether the microbial composition on the surface of the lesions differed from the microbes that were associated with the lesioned body wall tissues, as identified in the LBW group (Fig. 5). Results from Observed Species, Chao1, and ACE did not show significant differences between the microbiomes collected from LS compared to the LBW (Fig. 5A-C). Beta diversity showed that the LS samples clustered closely together compared to the LBW samples, which were less similar and more spread out with a large confidence interval (Fig. 5D). This indicated that the microbial composition among the LS samples was similar, and that there was more variation among the LBW samples. Furthermore, because the two clusters overlapped, the microbial compositions between the LS group and the LBW group likely had many shared taxa. The microbial composition in the sSW was very different from the samples collected from both the LS and the LBW. These results suggested that microbiome compositions were similar among the LS samples and less similar among the LBW samples, and were both different from the microbes in the aquarium seawater.

**Fig. 6:**
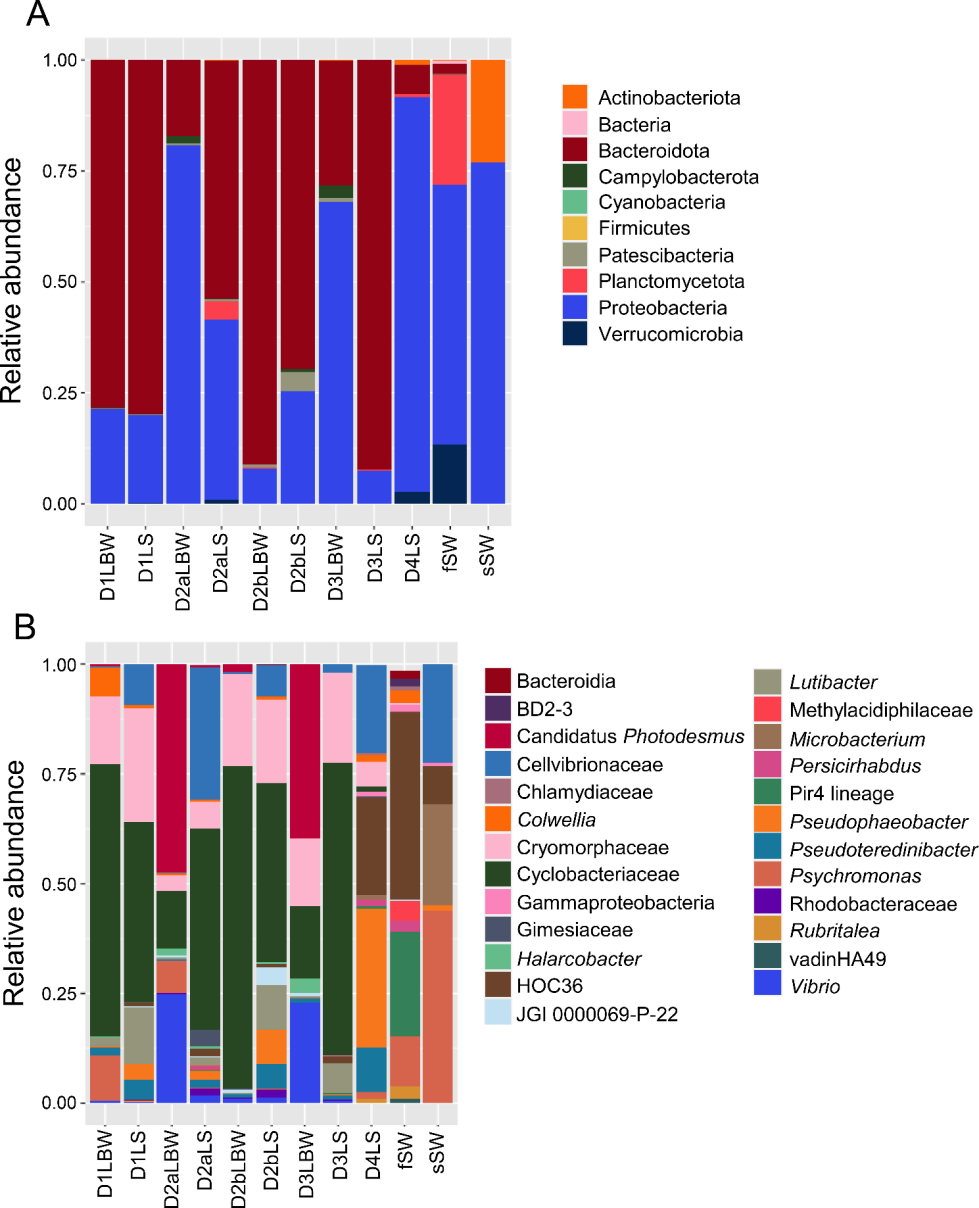
The microbiomes of the lesion surface and the lesioned body wall are highly similar. **A** All identified phyla are shown as the relative abundance in each sample (Additional File, Table S4). **B** Genera with an average relative abundance of > 0.1% across all groups (Additional File, Table S5) are illustrated by the relative abundance per sample. Abundance of taxa from replicated samples are averaged. Both types of seawater control samples are included for comparisons, which are the microbes collected from 500 ml of filtered seawater (fSW) and seawater collected with a swab (sSW). Taxa in **A** and **B** that could not be assigned at the level of phylum or genus are listed as the most specific known taxonomic level. Taxa that could not be assigned to a phylum are grouped under Bacteria. Sample name abbreviations are defined in Table 1. BD2-3 is in the order Victivallales, the Pir4 lineage is in the family Pirellulaceae, vadinHA49 is in the phylum Planctomycetota, JGI-0000069-P22 is in the class Gracilibacteria, and HOC36 is in the class Gammaproteobacteria

Further analysis of the microbiomes of the LS, LBW, and seawater samples (fSW and sSW) was carried out to compare the taxa identified in those sample groups (Fig. 6). At the level of phylum, the microbial composition of the LS and LBW microbiome samples were similar and were composed of mainly Proteobacteria and Bacteroidota, which varied inconsistently among the samples (Fig. 6A; Additional File, Table S4). At the level of genus, there were also many similarities in the microbial compositions (Fig. 6B; Additional File, Table S5), which were consistent with the beta diversity results (Fig. 5D). The LS samples from D1-D3 were highly similar to each other and were mainly dominated by a genus in the Cyclobacteriaceae family, a genus in the Cryomorphaceae family, and a genus in the Cellvibrionaceae family. The LS sample from D4, which had a single small lesion, differed from the others and was mainly composed of a genus in the Cellvibrionaceae family, the HOC36 group, and *Pseudophaeobacter*. The LBW samples also had an elevated abundance of a genus in the Cyclobacteriaceae family, and a genus in the Cryomorphaceae family, however, two of the LBW samples (D2a and D3) had elevated abundances of *Vibrio* and Candidatus *Photodesmus*. LEfSe results identified Cellvibrionaceae, *Pseudophaeobacter*, the HOC36 group, Gammaproteobacteria, and *Roseobacter* as significantly differentially abundant in the LS microbiome samples, whereas Canditatus *Photodesmus* was significantly differentially abundant in the LBW microbiome samples (Fig. 4B; Additional File, Table S6). Overall, all LBW samples were composed of a similar set of microbes.

When the LS microbiome samples from the diseased sea urchins were compared to each other, as well as to the sSW sample, D1-D3 had similarly abundant taxa, however, the LS microbiome from D4 differed from the other LS microbiomes (Fig. 6). Although an initial aim was to compare the microbiomes of the LS samples with non-lesioned surface areas of sea urchins with spotting disease, attempts to collect microbial gDNA from the surfaces of non-lesioned tissue using the swab method was unsuccessful. The 16S rRNA gene could not be amplified from the DNA isolated from the non-lesion surface swab samples indicating that were likely too few microbes associated with surfaces of healthy sea urchins, in agreement with a previous report [7]. Although another, more destructive collection method has been reported with more success for isolating bacterial gDNA from sea urchin surfaces and spines [24], this approach would not have been comparable to the LS sampling that was collected with swabs. Consequently, comparisons to non-lesioned surface microbiomes collected by the swab method could not be carried out.

Comparisons of the abundant taxa in the microbial compositions of the seawater samples showed that sSW sample included a genus in the Cellvibrionaceae family, *Microbacterium*, and *Psychromonas*. The abundant taxa in the fSW samples consisted mainly of the HOC36 group, the Pir4 lineage, and *Psychromonas*. Differences in the microbial composition of these seawater samples collected from the same aquarium may be attributed to differences in the volume of seawater collected. The small water volumes collected by swabs for the sSW samples may not have acquired an evenly distributed sample of microbes. Nonetheless, when the LS microbiomes were compared to the seawater samples, there were many taxa in the LS microbiomes that were absent from the microbiomes collected from either of the aquarium seawater samples. Overall, results indicated that the LS microbiome was distinct from the microbes in the seawater, but that it was highly similar to the LBW microbiome, which suggested similar a microbial composition on the surface of the lesion and within the lesioned body wall.

### Microbiomes are different between diseased and healthy sea urchin tissues

**Fig. 7:**
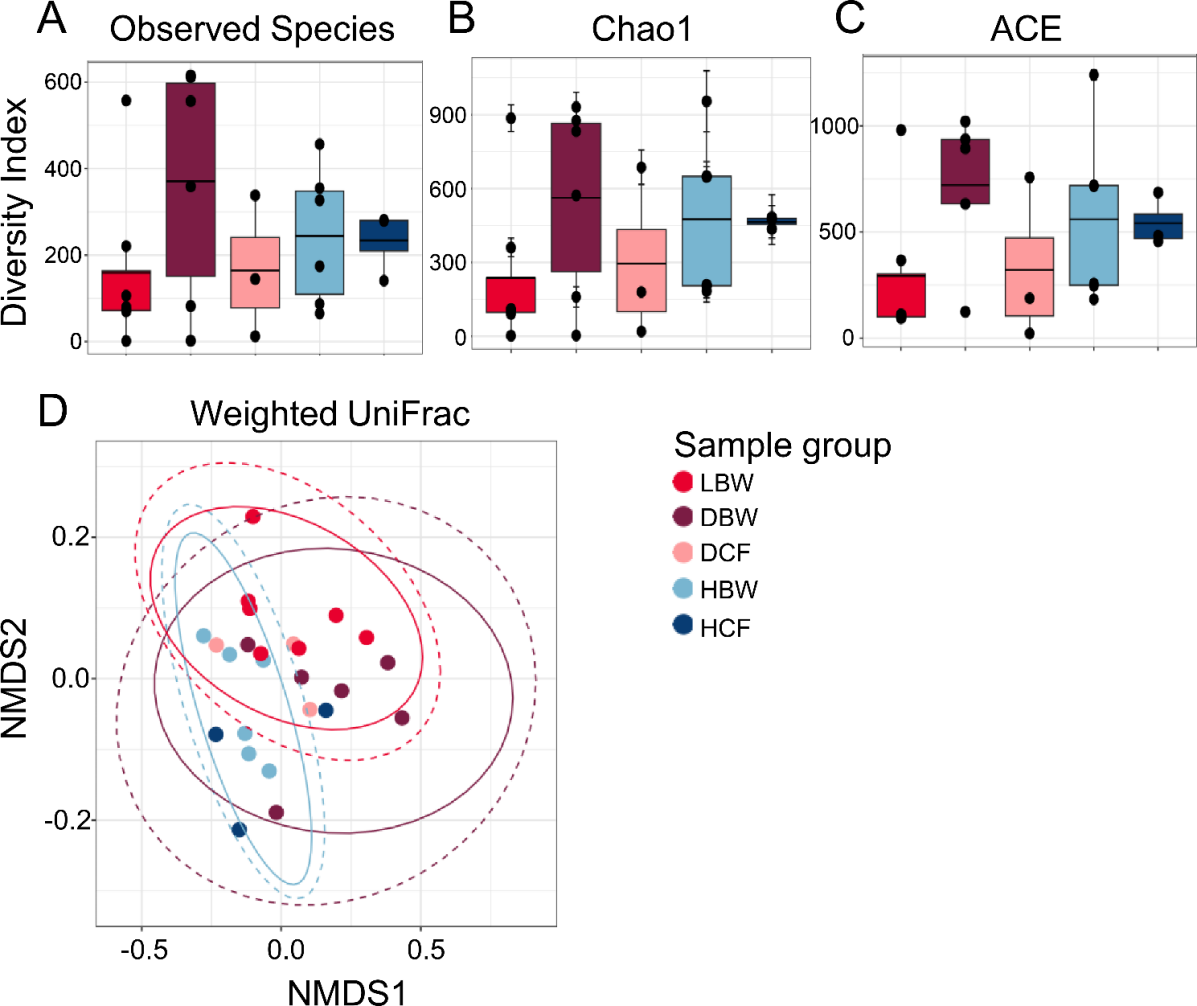
The microbiomes of dissected tissues from diseased and healthy sea urchins are different. Alpha diversity is analyzed by **A** Observed Species, **B** Chao1, and **C** ACE. The box plots show the mean and quartile values for each group, which are not significantly different (ANOVA, *p* > 0.05). **D** Beta diversity is analyzed at the ASV level using weighted UniFrac. Ellipses around sample groups show 95% confidence intervals assuming a multivariate t-distribution (solid line) and a multivariate normal distribution (dashed line). Groups are not significantly different (PERMANOVA, *p* > 0.05). Sample name abbreviations are defined in Table 1

A comparison of the microbiomes of dissected tissues from diseased compared to healthy sea urchins housed in the same closed aquarium system eliminates the variability of the microbial environment and is therefore informative for characterizing the microbiomes associated with the spotting disease infection on tissues that are outside of the lesioned areas. The alpha diversity metrics of the microbiomes from dissected tissues from infected sea urchins were evaluated and compared to each other and to the microbiomes of dissected tissues from healthy sea urchins (Fig. 7). Results from Observed Species, Chao1, and ACE, identified large variations in the samples within the groups of sea urchin tissues and therefore did not show significant differences among the groups (ANOVA, *p* > 0.05) (Fig. 7A-C). Beta diversity showed that the sample groups largely overlapped, but that the LBW samples tended to cluster together, and the HBW samples also clustered (Fig. 7D). The DBW samples had large variations, and therefore had a large confidence interval. The DCF samples tended to cluster with the LBW samples, whereas the HCF samples clustered with the HBW samples, indicating similar microbiome compositions for these pairs of sample groups. The beta diversity analysis revealed differing microbial compositions that suggested unique microbiomes for the LBW, DBW and HBW tissues.

**Fig. 8:**
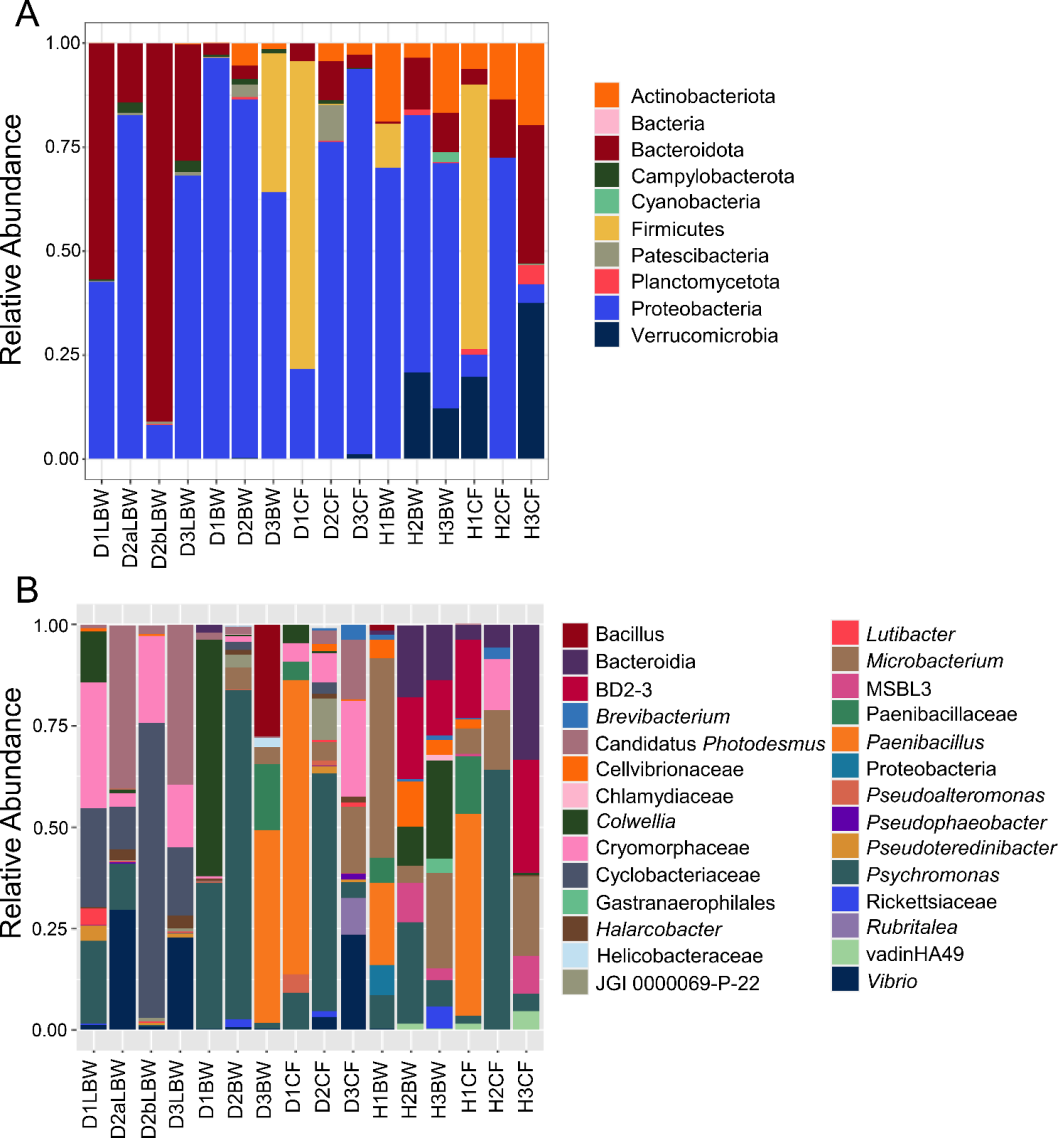
The microbiome of each dissected tissue has a distinct microbial composition. **A** All phyla that were identified are shown as the relative abundance of each phylum in each sample (Additional File, Table S7). Taxa that could not be assigned to a phylum are grouped under Bacteria. **B** Genera with an average relative abundance of > 0.1% across all groups are shown as their relative abundance per sample (Additional File, Table S8). Taxa that could not be assigned at the level of genus are listed as the most specific known taxonomic level. Sample name abbreviations are defined in Table 1. BD2-3 is in the order Victivallales, vadinHA49 is in the phylum Planctomycetota, JGI-0000069-P22 is in the class Gracilibacteria, and MSBL3 is in the family Kiritimatiellaceae

To understand the taxa underlying the bacterial compositions of the dissected tissues, the phyla identified in the microbiomes were compared among the samples (Fig. 8). Differences in the microbial composition were evident among the different tissues based on the relative abundance of the phyla (Fig. 8A; Additional File, Table S7). All groups were dominated by Proteobacteria, however, the LBW group including D1, D2a, b, and D3 (Fig. 1), was also largely composed of Bacteroidia, which was generally only present in low abundances in the other samples. The DBW samples from D1, D2, and D3 were largely composed of Proteobacteria with a few other phyla of low abundance, which was similar to the DCF samples from the same animals. The HBW group and HCF group from the healthy sea urchins similarly had low abundances of Bacteroidia, as well as Actinobacteriota and Verrucomicrobia that were not detected in the LBW samples. The taxa were also compared at the level of genus to identify differences among the sample groups (Fig. 8B; Additional File, Table S8). All LBW samples had an elevated abundance of a genus of the Cryomorphaceae family and a genus of the Cyclobacteriaceae family. In addition, there was an elevated abundance of Candidatus *Photodesmus* and *Vibrio* in two of the four LBW samples. The DBW samples differed in their microbial compositions, which was consistent with the large variation shown by beta diversity. The DCF samples, similar to the DBW samples, had differing microbial compositions for different sea urchins. The HBW samples consisted of a few taxa in common, which included *Psychromonas*, a genus of the Cellvibrionaceae family, *Colwellia*, and *Microbacterium*, among others. The microbial composition of the HCF samples differed based on the sea urchin, however, there were many taxa that were also present in the HBW samples, which included BD2-3, *Microbacterium*, *Psychromonas*, and a family of the Bacteroidia class.

Significant differential abundance of taxa for individual sample groups was identified by LEfSe analysis (Fig. 4C; Additional File, Table S9). Results showed that these taxa in the LBW samples were Candidatus *Photodesmus*, Cyclobacteriaceae, Cryomorphaceae, *Vibrio*, *Pseudoteredinibacter*, and *Lutibacter*. Taxa identified for the DCF samples were *Pseudoalteromonas* and *Pseudophaeobacter*, and taxa identified for the HCF samples were *Microbacterium*, BD2-3, Bacteroidia, vadinHA49, and *Brevibacterium*. Overall, the microbiome of the LBW was composed of a few taxa which were generally absent from the samples of the other groups. The microbiomes in the tissues from diseased sea urchins were distinct from those from healthy sea urchins, as evidenced by the differences between the DBW compared to the HBW samples, and the DCF compared to the HCF samples.

### Spotting disease and BSUD may have different etiologies

During this study and as reported previously [15], we observed differences between spotting disease and BSUD that both occurred in our aquaria. After receiving a shipment of sea urchins and placing them in aquarium B, all animals from that shipment contracted BSUD (see Fig. 1 in [15]). In comparison, there were four sea urchins (D1-4) that were housed in aquarium C that originated from a different shipment and became infected with spotting disease. BSUD was communicable based on the spread of the disease to all animals in aquarium B, including a number of sea urchins that were transferred into aquarium B during the outbreak [15]. D3, with a single discrete spotting disease lesion, was one of the sea urchins transferred into aquarium B with animals infected with BSUD. It began to show symptoms of BSUD about 60 days after the transfer including agitated spine movement, tissue swelling at the base of the spines, and primary spine loss, consistent with the other sea urchins with BSUD [15]. As a result, sea urchin D3 appeared to be infected with both BSUD and spotting disease simultaneously and the symptoms of each disease could be discerned separately. D3 did not succumb to BSUD but followed the same infection and recovery process as the other animals in the aquarium, including primary spine regrowth over the course of 2–3 months. However, the spotting disease lesion on D3 did not resolve. BSUD and spotting disease appeared as separate diseases based on their different symptoms and different recoveries, particularly for D3.

## Discussion

### The global surface microbiomes on diseased sea urchins are different from those on healthy sea urchins

The microbial composition of the global surface microbiomes for the healthy compared to the diseased sea urchins show differences. This is notable for sea urchins D1 and D2 that had the largest surface lesions, which is consistent with differences in microbial taxa between D1 and D2 compared to D3 and D4. For example, the lesion on D4 was much smaller than the lesions on the other infected sea urchins (Fig. 1), perhaps because it may have been more recently contracted, suggesting less impact of lesion-associated microbes on the global surface microbiome. The possibility of an early-stage infection on D4 may be a basis for the similarity of the bacterial composition with the surface microbiomes of the healthy sea urchins rather than the surface microbiomes for D1 and D2 (Fig. 3B). Furthermore, some of the key genera in the LS microbiomes for D1 and D2 are members of the Cryomorphaceae and Cyclobacteriacae families, which are also some of the key genera in their global surface microbiomes. This infers that the taxa present on the LS, which is the most proximal source of microbes to the entire animal surface, may influence or perhaps invade and become established in the microbial composition of the global surface microbiome of diseased sea urchins. There is a correlation between larger lesions and increased changes to the microbiome on the global surface of the animal. Taxonomic differences are evident between the diseased and healthy surface microbiomes despite factors that may reduce these differences, such as the microbes in the fSW sample and the varying stages of the lesions among the diseased sea urchins. These differences are especially noteworthy given that all sea urchins are housed in the same aquarium. These results indicate that the spotting disease infection alters the global surface microbiome of diseased sea urchins, which is different from the microbes in the seawater of the aquarium.

### The microbiomes of the lesion surface and the lesioned body wall are highly similar

The LS microbiome and the LBW microbiome are similar (Figs. 5 and 6) indicating that the microbes collected from the lesion surface and those identified from the entirety of the lesion including the body wall are largely uniform. This suggests that there is no surface biofilm composed of different taxa or a different relative composition of taxa. Among the diseased sea urchins, LS microbiome samples have similar microbial compositions (Fig. 5D), which is noteworthy considering that the compositions of the LBW microbiome samples are less similar. However, the LS of D4 (Fig. 6) is composed of different taxa than the other samples, which may be the outcome of the small lesion size that resulted in sampling difficulty. The similarities in the microbial composition between the LS and LBW microbiomes indicates that the same taxa are present throughout the lesion, and that each lesion has the same microbial composition for each of the diseased sea urchins. The identification of similar taxa based on two different methods of sampling strengthens the conclusion that these taxa underlie the spotting disease lesions.

### The microbiome of diseased sea urchin tissues differs from that of healthy sea urchin tissues

The microbiomes characterized from dissected tissues show that the samples from diseased sea urchins are different from samples from the healthy sea urchins. The HBW samples all have similar microbiomes, and therefore may be considered as an indicator of a standard microbial membership and composition for healthy sea urchins in our aquarium. However, it should be noted that a single “healthy” microbiome likely does not exist, as a major characteristic of a healthy microbiome is its ability to be dynamic [23]. Nonetheless, because the microbiomes of the DBW samples differ from those of the HBW samples (regions of non-lesioned tissue) from sea urchins in the same aquarium, this indicates that the spotting disease infection affects surface areas of the sea urchin that are outside of the observable lesions. This is also the case for the DCF for which the microbial composition differs from that of HCF. Although microbes have been shown to be present in the CF of sea urchins [35], another report shows that the coelomic fluid is sterile and lacks bacteria completely [36]. Differences may be based on methods of CF collection. For example, the body wall of sea urchins cannot be sterilized prior to CF collection by needle aspiration, therefore the presence of microbes in the coelomic fluid may be due to external contamination. Here, microbes are identified in the HCF samples, however, because of the sacrifice method, an unknown portion of the microbes in the CF samples are likely the result of contamination from other tissues. Sources may include the body wall, which must be cut through for collection, and/or from the esophagus - gut intersection that is broken when Aristotle’s lantern is removed prior to collecting the CF. The likelihood of contamination is supported by our results because the microbial composition of the HCF microbiome shares many similarities with the HBW microbiome samples (Fig. 8). Previous studies of the gut microbiome from *S. purpuratus* in various housing conditions have found that the typical composition is mainly Proteobacteria and Bacteroidetes, as well as *Vibrio*, *Arcobacter*, *Sulfurimonas*, *Desulfotalea*, *Psychromonas*, and *Shewanella* among others [37, 38]). Based on the microbial composition identified for the tissue microbiome samples reported here, there are many similarities between these taxa and the taxa reported previously for the gut microbiome in *S. purpuratus* [37, 38]. Because all sea urchins were housed in the same aquarium for years, this supports the notion that microbes from the gut that contaminated the CF samples would be similar for all dissected sea urchins. However, the microbial composition of the DCF is different from that of the HCF, suggesting that the spotting disease lesions introduce microbes into the coelomic fluid, which are different from gut contaminants, and that they are not cleared by coelomocytes or other attributes of the immune system. This is consistent with many shared microbes in the DCF samples with microbes in the LBW including Cryomorphaceae, *Vibrio*, and Candidatus *Photodesmus*. Consequently, the bacteria in the lesions that degrade and penetrate the test gain access to the coelomic fluid.

The taxa we identify that are present in the spotting disease lesions includes *Vibrio* that are consistently present for most or all samples of the LBW and are increased compared to healthy sea urchins, which is consistent with a previous report on spotting disease [5]. However, because culturing marine bacteria often yields only a small subset of the total microbes in environmental samples [39], we opted not to culture bacteria and therefore we did not perform experiments to confirm a causative agent based on Koch’s postulates. Nonetheless, the pathogenic bacteria that cause spotting disease may be normally associated with the tissues of sea urchins, and that they only become opportunistic pathogens upon colonizing wounds on the sea urchin surface. Based on these results and based on the wide array of bacteria that have been reported as causative agents of spotting disease, the disease may be the outcome of many different pathogenic bacteria, that may normally be associated with sea urchin tissues.

### The microbiome composition differs among reports of spotting disease infections

The microbial characterization associated with spotting disease reported here differs from previous reports. The bacteria associated with spotting disease are either based on identification of culturable bacteria [2, 7] or are identified through analysis of 16S rRNA gene sequences ([5], this study), and sometimes both [4]. There are multiple reports of BSUD and the associated bacteria, but that describe discrete lesions that are characteristic of spotting disease [8, 13, 19, 40]. Consequently, reports that include descriptions of discrete lesions will be considered here as spotting disease infections irrespective of the disease named in the paper. A variety of pathogenic bacteria cause or are associated with the discrete lesions, which include *Vibrio* sp [5, 40], *V. alginolyticus* [19], *V. coralliilyticus* [4], *Flexibacter* sp [2], *Acinetobacter* sp [7], *Tenacibaculum* sp [41], *Colwellia* sp, Flexibacteraceae, Rhodobacterales, *Stappia* [40], *Psychrobacter, Staphylococcus* [5], Saprospiraceae, and *Cohaesibacter gelatinilyticus* [13]. In this study, Cryomorphaceae, Cyclobacteriaceae, Candidatus *Photodesmus*, and *Vibrio* are the major taxa in the microbiomes of the spotting disease lesions on sea urchins in our aquarium. These taxa belong to either Bacteroidota or Gammaproteobacteria, but otherwise, there is little in common amongst the various bacteria that underlie the spotting disease infection. Species in the *Vibrio* genus appear to be generally associated with sea urchin diseases, including spotting disease [4–6, 11, 42, 43]. However, despite the discrete lesions on the body wall of infected sea urchins that have similar appearances across different species of sea urchins and in different locations in the oceans, the bacteria associated with the lesions are relatively diverse, spanning many different orders. The majority of taxa identified in this study have not been identified as pathogens, and may be commensals to echinoids. *Colwellia* has been isolated from seawater as well as a variety of marine organisms, which includes sea urchins [13]. It has been characterized as an opportunistic pathogen of the seas urchin, *Strongylocentrotus intermedius* [44], and therefore may be acting as a pathogen in this study. Cyclobacteriaceae have been isolated from seawater and echinoids, likely acting as commensals [45], although they have also been associated with diseased corals [46]. Cyclobacteriaceae belong to the bacterial order Cytophagales that are known for their ability to degrade chitin and other biopolymers such as pectin, cellulose, and agar, and may be involved in carbon cycling or remineralization of organic carbon [47–50]. While it is possible that Cyclobacteriaceae are actively involved in spotting disease progression, it may be more likely that they act as decomposers to degrade complex carbon compounds into simple units in marine environments. However, Cytophagales (and Rhodobacterales) also proliferate in coral tissues infected with *Vibrio coralliilyticus*, suggesting that they are opportunistic and act as destabilizers of the microbiome in diseased corals [51]. Thus, while the Cytophagales probably are not responsible for initiating spotting disease in echinoids, they may be involved in its progression. Cryomorphaceae have been isolated from diseased echinoids, however they have not been shown to be pathogenic [40]. It is more likely that they are opportunists or secondary pathogens along with Rhodobacteraceae, as in the case for corals [52, 53]. *Candidatus Photodesmus* is not well documented, and its functions in marine systems are unclear. The possibility that this bacterium is a commensal, pathogen, or both cannot be ruled out. Although *Vibrio* is involved in a variety of marine diseases, it also acts as a commensal and is an integral part of the sea urchin microbiome [5, 13, 38]. The *Vibrio splendidus* clade is associated with a disease affecting the sea urchin, *Paracentrotus lividus* [21], and *Vibrio coralliilyticus* has been identified as the primary agent of red spotting disease in *S. intermedius* [4]. Consequently, it is likely that it has important functions in spotting disease reported here. In addition to the taxa described above, many of those identified in *S. purpuratus* have been isolated from other marine organisms, suggesting functions as commensals in the echinoid microbiome. The host-microbe interaction is complex and commensals may become pathogenic in response to environmental changes. While limited information exists regarding the function of these specific taxa within the microbiota of marine organisms, the integration of our findings with the current literature suggests that spotting disease may be the result of, or in part due to, an overgrowth of commensal bacteria following a stressor that effects the host immune system. Our results add to this wide array of bacteria that are associated with spotting disease, and suggest that whether the spotting disease infection occurs in a closed aquarium system, in aquaculture facilities [2, 4, 5], or in natural environments [8, 19, 40], the putative pathogens are diverse. The taxa that invade tissues are likely the taxa that are present in or on the sea urchin tissues at the time of injury, which is assumed to be required to initiate a lesion [6, 7]. Overall, the integration of our results with previous reports suggests that the characteristic spotting disease lesions are likely caused by many different bacterial species, forming what is known as the “pathobiome”, which is a subset of microbes that is associated with negative effects on host health [54].

### The appearance and outcome of spotting disease is distinct from bald sea urchin disease

Spotting disease and BSUD are described in the literature interchangeably with a wide range of overlapping symptoms that lead to confusions in distinguishing the diseases [1, 11, 15]. Based on our observations of an outbreak of BSUD in aquarium B, and separate infections of spotting disease in aquaria B and C, as well as sea urchin D3 that was infected with both BSUD and spotting disease simultaneously, we conclude that spotting disease and BSUD are separate diseases based on different symptoms, infectivity, severity, and lethality. The spotting disease infection that we observed is consistent with other reports of the disease, based on the descriptions of discrete surface lesions [1, 5]. The BSUD infection that we report [15], however, is different from other reports of BSUD that include descriptions of discrete lesions [7, 8, 19], which our sea urchins do not show. Our observations suggest that whole-body surface infection vs. discrete lesions are defining characteristics of BSUD vs. spotting disease, respectively, because lesions are present in all cases of spotting disease but are not necessarily a symptom of BSUD. In contrast, an infection that encompasses the entire animal surface and causes partial to complete loss of surface appendages is the key symptom of BSUD, but not spotting disease. The confusions in the literature may be based on the possibility that sea urchins can be infected simultaneously with both diseases ([4, 21], this study). Furthermore, when sea urchins infected with both diseases in our aquaria were treated with pen/strep; those with BSUD recover [15], however, those with spotting disease do not. The different outcomes from the antibiotic treatment suggest distinct characteristics that may be due to differences in the microbes that underlie the diseases. Based on the descriptions in the literature, there are reports of sea urchin diseases that have been identified as BSUD but should be classified as spotting disease because they describe discrete lesions [6, 7, 19, 20, 55].

### The microbial composition associated with spotting disease is distinct from BSUD

Evaluation of the global surface microbiome associated with spotting disease employed the same approaches and methods to evaluate sea urchins in the same aquarium that had BSUD [15]. Consequently, the global surface microbiomes associated with the two diseases can be compared. There is no single taxon that dominates the microbiome composition on sea urchins with BSUD, and consequently, a combination of many taxa likely underlie the infection. Although spotting disease and BSUD have distinct symptoms, the genus *Lutibacter* is associated with both, suggesting that when sea urchins become compromised, this may be a taxon that is generally prevalent and is therefore likely to invade. *Lutibacter* belongs to the Flavobacteriaceae family (class Flavobacteriia) that is related to the Cytophagia class in a group previously known as Cytophaga, Flavobacteria, Bacteroides or CFB [56]. These bacteria are commonly found in marine habitats and are associated with certain species of sea urchins [57, 58]. Some members of the Flavobacteriaceae family are known pathogens, such as *Flavobacterium psychrophilum* that infects salmonids [59, 60]. The Flavobacteriaceae frequently express genes encoding proteases and proteins involved in degrading complex biopolymers, as well as virulence factors for host invasion [61]. It is notable that bacteria closely related to *Lutibacter litoralis* have been isolated from an American lobster (*Homarus americanus*) with ulcerative enteritis [62]. Therefore, we speculate that although *Lutibacter* spp may be commensals associated with sea urchins, they may have a propensity to degrade biopolymers that are found in these animals. Although *Colwellia* is elevated in the global surface microbiomes of the sea urchins infected with BSUD, in this report it is associated with the healthy sea urchins, suggesting its involvement mainly in BSUD and less in spotting disease. The variations in the taxa associated with these two diseases also suggests that different combinations of bacteria may underpin variations in the symptoms. This may be based on which bacteria become the dominant taxa, and how the proliferative opportunity arises; wound infection for spotting disease vs. altered microbial dynamics for BSUD. It is noteworthy that when the sea urchins recover from BSUD their surface microbiomes change and closely resemble the microbial composition of the fSW [15]. However, the microbial composition of the surface samples from the sea urchins with spotting disease do not share many similarities with the microbes in the fSW samples.

Reproducing discrete spotting disease lesions experimentally requires both surface abrasion and the introduction of microbes into the damaged tissue [7, 8]. Lesions do not develop in the absence of surface abrasion suggesting that both abrasion and microbial infection are required for the development of lesions. Unlike naturally occurring spotting disease however, sea urchins used for experimental induction of spotting disease typically resolve the lesions and recover [7, 8]. Although the appearance of the experimentally induced lesions are similar to naturally derived lesions, the recovery from spotting disease in experimentally induced infections compared to infections that appear in nature suggests that there may be other parameters in play that are required to result in spotting disease in addition to tissue damage. These may include (i) physiological stresses impacting sea urchin defense functions, (ii) differences in the bacterial composition of the species introduced into the injuries, and/or (iii) differences in the microbiomes on the global surface of the experimental sea urchins in aquaria compared to those in nature, which may impact or compete with the microbes that may invade the damaged tissue. Sea urchins infected with spotting disease in natural settings may be exposed to a greater quantity, concentration, or variety of opportunistic taxa that may exacerbate the pathogenicity. Sea urchins in different ecological habitats or in different aquarium systems also show significant differences in their global surface microbiomes [15, 24]. However, in this study, unlike experimentally induced spotting disease, the sea urchins that contracted lesions naturally while in a closed system did not resolve their lesions and did not recover. This suggests that the sea urchins in our aquaria were stressed physiologically, which likely compromises the immune response thereby releasing the microbiomes associated with the sea urchins from immunological control. The outcome may have been the proliferation and invasion of pathogenic bacteria into tissues rather than being restricted to the surface, which led to the development of focal lesions that could not be resolved. *Strongylocentrotus purpuratus* is commonly found in large clusters in nature that are necessary for successful spawning events and for communal capture of large drift kelp (e.g., see Fig. 19.2c in [63]). Injuries from spines among closely neighboring animals that compete for food may result in ongoing minor injuries that may normally resolve. The basis for whether the injuries progress to lesions that do not resolve under natural settings is likely an indication of poor condition of the sea urchins perhaps resulting from environmental stressors.

## Conclusions

Here, we characterize the microbiomes associated with various tissues from sea urchins infected with spotting disease and housed in a closed aquarium. We show that the tissues of diseased and healthy sea urchins have distinct microbiomes, and that the spotting disease infection affects areas of the body wall and surface tissue outside of the lesioned area as well as the coelomic fluid. The combination of surface injuries, microbial infections of the injuries, and a compromised defense system, perhaps as an outcome of physiological stress in sea urchins, combine to result in spotting disease. The characteristic lesions of spotting disease are dominated by Cyclobacteriaceae, Cryomorphaceae, and a few other taxa, which may be the underlying pathogens, which invade injuries on healthy sea urchin tissues that progress to tissue necrosis. The key symptom of spotting disease is discrete surface lesions with restricted surface appendage loss whereas BSUD is a distinct disease with the key symptom of a global surface infection resulting in general spine loss.

### Electronic supplementary material

Below is the link to the electronic supplementary material.

**Supplementary Material 1: Table S1.** Relative abundance of phyla on the global surface microbiome samples. **Table S2.** Genera with an average relative abundance > 0.1% shown as the relative abundance per sample for the global surface microbiome samples. **Table S3.** Significantly differentially abundant taxa as identified by LEfSe for diseased and healthy global surface microbiome samples. **Table S4.** Relative abundance of phyla for the LBW and LS samples. **Table S5.** Genera with an average relative abundance of > 0.1% in the LBW and LS microbiome samples. **Table S6.** Significantly differentially abundant taxa as identified by LEFSe for the LBW and LS microbiome samples. **Table S7.** Relative abundance of phyla in the tissue microbiome samples. **Table S8.** Genera with an average relative abundance of > 0.1% in the tissue microbiome samples. **Table S9.** Significantly differentially abundance taxa as identified by LEfSe in the tissue microbiome samples

## Data Availability

The raw sequence reads are available in the Sequence Read Archive database at NCBI under the BioProject ID PRJNA937707. The R code with the complete pipeline is available in the GitHub repository under Spotting_disease_S_purpuratus.
