# Supplementary material for: Spotting disease disrupts the microbiome of infected purple sea urchins, Strongylocentrotus purpuratus
Source: BMC Microbiol. 2024 Jan 4;24:11. doi: 10.1186/s12866-023-03161-9 (PMC10765733; doi:10.1186/s12866-023-03161-9)
Supplement: Supplementary file 1 — Supplementary Material 1: Table S1. Relative abundance of phyla on the global surface microbiome samples. Table S2. Genera with an average relative abundance > 0.1% shown as the relative abundance per sample for the global surface microbiome samples. Table S3. Significantly differentially abundant taxa as identified by LEfSe for diseased and healthy global surface microbiome samples. Table S4. Relative abundance of phyla for the LBW and LS samples. Table S5. Genera with an average relative abundance of > 0.1% in the LBW and LS microbiome samples. Table S6. Significantly differentially abundant taxa as identified by LEFSe for the LBW and LS microbiome samples. Table S7. Relative abundance of phyla in the tissue microbiome samples. Table S8. Genera with an average relative abundance of > 0.1% in the tissue microbiome samples. Table S9. Significantly differentially abundance taxa as identified by LEfSe in the tissue microbiome samples [file 12866_2023_3161_MOESM1_ESM.pdf]

Additional File

Spotting disease disrupts the microbiome of infected sea urchins

Chloe G. Shaw, Christina Pavloudi, Ryley S. Crow, Jimmy H. Saw, L. Courtney Smith

Supplementary Tables

Table S1. Relative abundance of phyla on the global surface microbiome samples

Table S2. Genera with an average relative abundance > 0.1% shown as the relative abundance per sample for the global surface microbiome samples

Table S3. Significantly differentially abundant taxa as identified by LEfSe for diseased and healthy global surface microbiome samples

Table S4. Relative abundance of phyla for the LBW and LS samples

Table S5. Genera with an average relative abundance of > 0.1% in the LBW and LS microbiome samples

Table S6. Significantly differentially abundant taxa as identified by LEFSe for the LBW and LS microbiome samples

Table S7. Relative abundance of phyla in the tissue microbiome samples

Table S8. Genera with an average relative abundance of > 0.1% in the tissue microbiome samples

Table S9. Significantly differentially abundance taxa as identified by LEfSe in the tissue microbiome samples

Table S1. Relative abundance of phyla on the global surface microbiome samples[1]

Phylum	D1S	D2S	D3S	D4S	H1S	H2S	H3S	H4S	fSW[2]
Verrucomicrobiota	2.51[3]	5.53	13.19	7.33	35.49	16.62	4.83	4.14	13.31
Planctomycetota	1.13	0.97	5.37	4.62	7.92	10.98	1.71	2.36	24.68
Bacteroidota	53.32	42.19	5.37	61.12	10.69	10.39	0.47	2.51	2.40
Proteobacteria	42.66	33.89	75.75	26.37	43.69	60.53	92.72	90.41	58.63
Patescibacteria	0.38	15.35	0.00	0.25	0.78	1.19	0.23	0.18	0.13
Actinobacteriota	0.00	0.00	0.24	0.12	0.00	0.00	0.00	0.03	0.15
Firmicutes	0.00	0.00	0.00	0.00	0.00	0.00	0.00	0.00	0.00
Bacteria	0.00	0.97	0.08	0.18	0.11	0.15	0.00	0.03	0.70
Campylobacterota	0.00	1.11	0.00	0.00	0.17	0.00	0.04	0.12	0.00
Cyanobacteria	0.00	0.00	0.00	0.00	1.16	0.15	0.00	0.21	0.00

[1]Abbreviations are: D, diseased animals 1-4; H, healthy animals 1-4; S; Surface, global surface microbiome samples. See also Table 1 in the main paper.

[2]Average of the taxa identified by filtering two sea water samples, fSW1 and fSW2.

[3]Percent relative abundance per sample.

Table S2. Genera with an average relative abundance > 0.1% shown as the relative abundance per sample for the global surface microbiome samples

**Species**	**D1S[1]**	**D2S**	**D3S**	**D4S**	**H1S**	**H2S**	**H3S**	**H4S**	**fSW[1]**
Psychromonas	4.77[2]	5.39	41.86	16.02	26.52	33.38	53.99	86.80	11.44
HOC36	29.86	4.15	6.08	3.14	7.09	2.67	2.06	0.98	42.76
Colwellia	1.51	9.41	22.20	5.98	8.53	23.15	34.60	1.96	2.66
Bacteroidia	1.51	4.29	3.48	59.77	10.30	9.94	0.47	2.51	1.83
Pir4 lineage	0.88	0.97	5.29	4.62	3.93	6.38	1.71	1.62	23.67
Cyclobacteriaceae	19.45	26.42	0.39	0.00	0.00	0.00	0.00	0.00	0.00
BD2-3	0.50	0.83	1.42	5.48	23.98	5.34	0.23	1.59	1.53
Cryomorphaceae	24.72	5.81	1.26	0.00	0.39	0.30	0.00	0.00	0.57
Methylacidiphilaceae	1.76	3.32	0.63	0.18	1.77	3.12	1.79	0.52	4.59
JGI 0000069-P22	0.38	15.35	0.00	0.25	0.78	1.19	0.23	0.18	0.13
Rubritalea	0.25	0.69	3.55	0.55	0.83	2.67	1.28	0.92	2.91
Persicirhabdus	0.00	0.00	6.08	0.31	1.22	1.63	1.25	0.55	2.52
MSBL3	0.00	0.69	1.26	0.37	7.36	3.56	0.19	0.55	0.57
Lutibacter	7.65	5.67	0.24	0.00	0.00	0.00	0.00	0.00	0.00
vadinHA49	0.25	0.00	0.00	0.00	3.99	4.60	0.00	0.74	1.00
Cellvibrionaceae	3.64	5.67	1.18	0.00	0.39	0.00	0.00	0.00	0.00
Pseudoalteromonas	0.00	0.00	2.92	0.06	0.50	0.74	1.60	0.31	0.22
Gammaproteobacteria	1.13	0.28	0.32	0.12	0.33	0.30	0.23	0.12	1.55
Pseudoteredinibacter	0.88	4.01	0.47	0.00	0.00	0.00	0.00	0.00	0.00
Pseudophaeobacter	0.63	2.77	0.32	0.00	0.06	0.00	0.00	0.00	0.00
Chlamydiaceae	0.00	0.00	0.24	0.43	0.33	0.30	0.08	0.00	1.19
Bacteria[3]	0.00	0.97	0.08	0.18	0.11	0.15	0.00	0.03	0.70
Candidatus *Photodesmus*	0.13	0.41	0.00	0.80	0.06	0.15	0.00	0.03	0.00
Gastranaerophilales	0.00	0.00	0.00	0.00	1.16	0.15	0.00	0.21	0.00
Bacteroidales	0.00	0.00	0.00	1.36	0.00	0.15	0.00	0.00	0.00
Rhodobacteraceae	0.13	1.24	0.00	0.00	0.00	0.00	0.04	0.03	0.00
Halarcobacter	0.00	1.11	0.00	0.00	0.17	0.00	0.04	0.12	0.00
Vibrio	0.00	0.55	0.16	0.25	0.00	0.00	0.08	0.00	0.00

[1]See Table S1 for definitions of abbreviations and Table 1 in the main paper.
[2]Percent relative abundance per sample.
[3]Bacteria denotes taxa that could not be assigned to a phylum.

Table S3. Significantly differentially abundant taxa as identified by LEfSe for diseased and healthy global surface microbiome samples

Taxa	Enriched group	LDA score[1]	*p* value	*p* adjusted
Gastranaerophilales	Healthy	3.54322854	0.0472209	0.0472209
Enterobacterales	Healthy	3.18015404	0.03839358	0.03839358
Cyclobacteriaceae	Diseased	5.09216384	0.0472209	0.0472209
Lutibacter	Diseased	4.57012195	0.0472209	0.0472209
Pseudoteredinibacter	Diseased	4.16907426	0.0472209	0.0472209

[1]The linear discriminant analysis (LDA) threshold was set to 2.

Table S4. Relative abundance of phyla for the LBW and LS samples

Phylum	D1 LBW[1]	D2a LBW	D2b LBW	D3 LBW	D1 LS	D2a LS	D2b LS	D3 LS	D4 LS	sSW[1]	fSW[1]
Verrucomicrobiota	0.01[2]	0.00	0.04	0.00	0.24	0.88	0.00	0.00	2.71	0.00	13.31
Planctomycetota	0.01	0.00	0.22	0.00	0.00	4.05	0.00	0.22	0.81	0.00	24.68
Bacteroidota	78.42	17.25	91.17	28.03	79.86	53.63	69.61	92.30	6.50	0.00	2.40
Proteobacteria	21.36	80.77	7.88	68.17	19.62	40.66	25.43	7.48	88.89	76.99	58.63
Patescibacteria	0.07	0.44	0.69	0.71	0.24	0.41	4.24	0.00	0.00	0.00	0.13
Actinobacteriota	0.03	0.00	0.00	0.24	0.00	0.05	0.00	0.00	1.08	23.01	0.15
Firmicutes	0.00	0.00	0.00	0.00	0.00	0.00	0.00	0.00	0.00	0.00	0.00
Bacteria[3]	0.00	0.00	0.00	0.00	0.00	0.00	0.00	0.00	0.00	0.00	0.70
Campylobacterota	0.10	1.54	0.00	2.85	0.05	0.31	0.72	0.00	0.00	0.00	0.00
Cyanobacteria	0.00	0.00	0.00	0.00	0.00	0.00	0.00	0.00	0.00	0.00	0.00

[1]Definitions of sample name abbreviations can be found in Table 1 in the main paper and Table S1.
[2]Percent relative abundance per sample.
[3]Bacteria denotes taxa that could not be assigned to a phylum.

Table S5. Genera with an average relative abundance of > 0.1% in the LBW and LS microbiome samples

Species	D1 LBW[1]	D2a LBW	D2b LBW	D3 LBW	D1LS	D2aLS	D2bLS	D3LS	D4LS	sSW	fSW[2]
Cyclobacteriaceae	62.23[3]	13.24	73.55	16.62	41.22	45.97	40.57	66.70	1.11	0.00	0.00
Cryomorphaceae	15.43	3.49	20.83	15.40	25.80	5.92	19.07	20.45	5.57	0.00	0.57
Candidatus *Photodesmus*	0.39	47.45	1.88	39.65	0.00	0.47	0.16	0.00	0.00	0.00	0.00
HOC36	0.03	0.00	0.05	0.00	0.67	1.77	0.57	1.70	22.28	8.52	42.76
Psychromonas	10.29	7.28	0.09	0.00	0.14	0.21	0.10	0.11	1.39	43.75	11.44
Cellvibrionaceae	0.38	0.19	0.40	0.00	9.19	30.08	7.08	1.93	20.06	22.44	0.00
Vibrio	0.64	24.96	0.99	22.75	0.19	1.77	1.29	0.57	0.00	0.00	0.00
Pir4 lineage	0.01	0.00	0.00	0.00	0.00	0.26	0.00	0.23	0.56	0.00	23.67
Pseudophaeobacter	0.10	0.05	0.00	0.00	3.54	2.08	8.06	0.34	31.48	1.42	0.00
Lutibacter	2.03	0.56	0.32	0.14	12.88	1.82	9.92	6.82	0.00	0.00	0.00
Pseudoteredinibacter	1.81	0.10	0.69	1.09	4.64	1.71	5.53	0.91	10.31	0.00	0.00
Microbacterium	0.03	0.00	0.00	0.27	0.00	0.05	0.00	0.00	1.11	23.01	0.07
Colwellia	6.37	0.41	0.00	0.00	0.86	0.57	0.67	0.00	1.67	0.00	2.66
Methylacidiphilaceae	0.00	0.00	0.00	0.00	0.00	0.16	0.00	0.00	0.00	0.00	4.59
JGI 0000069-P22	0.07	0.44	0.71	0.82	0.24	0.42	4.24	0.00	0.00	0.00	0.13
Halarcobacter	0.11	1.55	0.00	3.27	0.05	0.31	0.72	0.00	0.00	0.00	0.00
Rubritalea	0.01	0.00	0.00	0.00	0.24	0.10	0.00	0.00	1.11	0.00	2.91
Persicirhabdus	0.00	0.00	0.00	0.00	0.00	0.62	0.00	0.00	1.39	0.00	2.52
Gammaproteobacteria	0.00	0.00	0.00	0.00	0.05	0.00	0.00	0.11	1.11	0.85	1.55
Rhodobacteraceae	0.00	0.24	0.21	0.00	0.19	1.56	1.86	0.11	0.00	0.00	0.00
Gimesiaceae	0.00	0.00	0.23	0.00	0.00	3.79	0.00	0.00	0.28	0.00	0.00
Bacteroidia	0.00	0.00	0.00	0.00	0.00	0.00	0.05	0.00	0.00	0.00	1.83
BD2-3	0.00	0.00	0.00	0.00	0.00	0.00	0.00	0.00	0.00	0.00	1.53
Chlamydiaceae	0.00	0.00	0.04	0.00	0.00	0.00	0.00	0.00	0.28	0.00	1.19
vadinHA49	0.00	0.00	0.00	0.00	0.00	0.00	0.00	0.00	0.00	0.00	1.00

[1]Definitions of sample name abbreviations can be found in Table 1 in the main paper and Table S1.
[2]Average of the taxa identified by filtering two sea water samples, fSW1 and fSW2.
[3]Percent relative abundance per sample.

Table S6. Significantly differentially abundant taxa as identified by LEFSe for the LBW and LS microbiome samples

**Taxa**	**Enriched group**	**LDA score**[1]	***p* value**	***p* adjusted**
Cellvibrionaceae	LS[2]	5.17687914	0.00255609	0.00255609
Pseudophaeobacter	LS	4.81626803	0.00202884	0.00202884
HOC36	LS	4.70352662	0.00202884	0.00202884
Gammaproteobacteria	LS	3.55714684	0.0145743	0.0145743
Roseobacter	LS	3.27102339	0.0145743	0.0145743
Candidatus *Photodesmus*	LBW[3]	5.19269004	0.01248356	0.01248356

[1]The linear discriminant analysis (LDA) threshold was set to 2.
[2]LS; Lesion surface samples.
[3]LBW; Lesion body wall samples.

Table S7. Relative abundance of phyla in the tissue microbiome samples

Phylum	D1[1] LBW	D2a LBW	D2b LBW	D3 LBW	D1 BW	D2 BW	D3 BW	D1 CF	D2 CF	D3 CF	H1 BW	H2 BW	H3 BW	H1 CF	H2 CF	H3 CF
Verrucomicrobiota	0.03[2]	0.00	0.04	0.00	0.06	0.25	0.00	0.00	0.00	1.25	0.01	20.80	12.34	19.87	0.00	37.41
Planctomycetota	0.03	0.00	0.20	0.00	0.00	0.42	0.00	0.00	0.19	0.00	0.00	1.13	0.19	1.47	0.00	4.70
Bacteroidota	56.86	14.32	90.93	28.03	2.68	3.30	0.00	4.35	9.49	3.32	0.43	12.51	9.40	3.70	13.91	33.36
Proteobacteria	42.70	82.78	8.16	68.17	96.36	86.28	64.16	21.74	76.28	92.56	70.12	62.08	58.83	5.17	72.61	4.70
Patescibacteria	0.13	0.48	0.67	0.71	0.10	3.02	0.00	0.00	8.81	0.00	0.00	0.00	0.00	0.00	0.00	0.05
Actinobacteriota	0.05	0.00	0.00	0.24	0.10	5.45	1.51	0.00	4.26	2.68	18.97	3.49	16.78	6.35	13.48	19.69
Firmicutes	0.00	0.00	0.00	0.00	0.12	0.00	33.47	73.91	0.10	0.00	10.46	0.00	0.00	63.43	0.00	0.00
Bacteria	0.00	0.00	0.00	0.00	0.00	0.00	0.00	0.00	0.00	0.00	0.00	0.00	0.00	0.00	0.00	0.00
Campylobacterota	0.21	2.41	0.00	2.85	0.59	1.29	0.87	0.00	0.87	0.19	0.00	0.00	0.00	0.01	0.00	0.10
Cyanobacteria	0.00	0.00	0.00	0.00	0.00	0.00	0.00	0.00	0.00	0.00	0.00	0.00	2.45	0.00	0.00	0.00

[1]Definitions of sample name abbreviations can be found in Table 1 in the main paper and Table S1.
[2]Percent relative abundance per sample.

Table S8. Genera with an average relative abundance of > 0.1% in the tissue microbiome samples

**Species**	**D1 LBW**[1]	**D2a LBW**	**D2b LBW**	**D3 LBW**	**D1 BW**	**D2 BW**	**D3 BW**	**H1 BW**	**H2 BW**	**H3 BW**	**D1 CF**	**D2 CF**	**D3 CF**	**H1 CF**	**H2 CF**	**H3 CF**
Psychromonas	20.57[2]	11.40	0.08	0.00	36.17	81.01	1.73	8.55	24.95	6.47	9.09	58.84	3.93	1.87	64.00	4.08
Paenibacillus	0.00	0.00	0.00	0.00	0.12	0.00	47.40	20.33	0.00	0.00	72.73	0.11	0.00	50.02	0.00	0.00
Microbacterium	0.05	0.00	0.00	0.27	0.10	5.43	4.11	49.21	4.34	23.59	0.00	4.45	16.29	6.17	14.86	19.66
Cyclobacteriaceae	24.51	10.59	73.01	16.62	0.00	2.01	0.00	0.00	0.00	0.00	0.00	3.00	0.00	0.00	0.00	0.00
Cryomorphaceae	30.85	3.56	21.30	15.40	0.65	1.33	0.00	0.00	0.00	0.00	4.55	7.12	23.60	0.00	12.57	0.00
Colwellia	12.73	0.65	0.00	0.00	58.37	0.34	0.00	0.18	9.79	23.94	4.55	0.56	0.00	0.00	0.00	0.49
Candidatus *Photodesmus*	0.79	40.50	1.98	39.65	1.59	1.53	0.22	0.00	0.00	0.00	0.00	3.67	14.89	0.00	0.00	0.05
Vibrio	1.28	29.59	1.03	22.75	0.14	0.57	0.22	0.22	0.00	0.04	0.00	3.34	23.31	0.02	0.00	0.05
BD2-3	0.00	0.00	0.00	0.00	0.00	0.00	0.00	0.02	20.16	13.62	0.00	0.00	0.00	19.40	0.00	27.86
Bacteroidia	0.00	0.00	0.00	0.00	2.04	0.00	0.00	1.15	17.63	13.82	0.00	0.00	0.00	3.74	5.71	33.28
Paenibacillaceae	0.00	0.00	0.00	0.00	0.00	0.00	16.23	6.14	0.00	0.00	4.55	0.00	0.00	14.00	0.00	0.00
Bacillus	0.00	0.00	0.00	0.00	0.00	0.00	27.71	1.33	0.00	0.00	0.00	0.00	0.00	0.11	0.00	0.00
Cellvibrionaceae	0.76	0.08	0.37	0.00	0.00	0.00	0.00	4.52	11.02	3.56	0.00	1.56	0.28	2.24	0.00	0.00
MSBL3	0.00	0.00	0.00	0.00	0.06	0.00	0.00	0.00	9.59	2.85	0.00	0.00	0.00	0.69	0.00	9.55
JG1 0000069-P22	0.14	0.49	0.70	0.82	0.10	3.06	0.00	0.00	0.00	0.00	0.00	10.12	0.00	0.00	0.00	0.05
Halarcobacter	0.22	2.43	0.00	3.27	0.59	1.27	0.00	0.00	0.00	0.00	0.00	1.00	1.40	0.01	0.00	0.10
Brevibacterium	0.00	0.00	0.00	0.00	0.00	0.08	0.00	1.19	0.65	1.08	0.00	0.44	3.65	0.25	2.86	0.03
Rubritalea	0.03	0.00	0.00	0.00	0.00	0.00	0.00	0.00	0.00	0.00	0.00	0.00	9.27	0.00	0.00	0.00
Rickettsiaceae	0.11	0.00	0.00	0.00	0.00	2.21	0.00	0.00	0.00	5.48	0.00	1.22	0.00	0.00	0.00	0.00
vadinHA49	0.00	0.00	0.00	0.00	0.00	0.00	0.00	0.00	1.62	0.28	0.00	0.00	0.00	1.49	0.00	4.70
Pseudoteredinibacter	3.62	0.16	0.70	1.09	0.00	0.00	0.00	0.00	0.00	0.00	0.00	1.45	0.56	0.00	0.00	0.00
Proteobacteria	0.00	0.00	0.00	0.00	0.00	0.00	0.00	7.17	0.00	0.00	0.00	0.00	0.00	0.00	0.00	0.00
Lutibacter	4.05	0.24	0.33	0.14	0.00	0.00	0.00	0.00	0.00	0.00	0.00	0.78	1.12	0.00	0.00	0.00
Pseudoalteromonas	0.00	0.00	0.00	0.00	0.10	0.25	0.00	0.00	0.00	0.00	4.55	1.22	0.28	0.00	0.00	0.00

[1]Definitions of sample name abbreviations can be found in Table 1 in the main paper and Table S1.
[2]Percent relative abundance per sample.

Table S9. Significantly differentially abundance taxa as identified by LEfSe in the tissue microbiome samples

Taxa	Enriched group	LDA score[1]	*p* value	*p* adjusted
Pseudoalteromonas	DCF[2]	3.90070717	0.00255785	0.00255785
Pseudophaeobacter	DCF	3.2835783	0.04218883	0.04218883
Microbacterium	HCF	4.91327528	0.00841746	0.00841746
BD2-3	HCF	4.89332032	0.0094217	0.0094217
Bacteroidia	HCF	4.84050663	0.01834497	0.01834497
vadinHA49	HCF	3.98707961	0.04004801	0.04004801
Brevibacterium	HCF	3.65176359	0.00252176	0.00252176
Cyclobacteriaceae	LBW	5.32451733	0.0006317	0.0006317
Candidatus *Photodesmus*	LBW	4.83418387	0.01436621	0.01436621
Cryomorphaceae	LBW	4.76870085	0.00726085	0.00726085
Vibrio	LBW	4.57190404	0.0312891	0.0312891
Pseudoteredinibacter	LBW	3.60968065	0.00872441	0.00872441
Lutibacter	LBW	3.55196684	0.00872441	0.00872441

[1]The linear discriminant analysis (LDA) threshold was set to 2.

[2]Definitions of sample name abbreviations can be found in Table 1 in the main paper. For this table, definitions are: D, diseased; H, healthy; CF, coelomic fluid; LBW, lesion body wall.
